# Colorectal cancer in patients with SARS-CoV-2: a systematic review and meta-analysis

**DOI:** 10.1186/s13027-022-00459-7

**Published:** 2022-09-12

**Authors:** Saad Alhumaid, Abbas Al Mutair, Jawad S. Busubaih, Nourah Al Dossary, Murtadha Alsuliman, Sarah A. Baltyour, Ibrahim Alissa, Hassan I. Al Hassar, Noor A. Al Aithan, Hani A. Albassri, Suliman A. AlOmran, Raed M. ALGhazal, Ahmed Busbaih, Nasser A. Alsalem, Waseem Alagnam, Mohammed Y. Alyousef, Abdulaziz U. Alseffay, Hussain A. Al Aish, Ali Aldiaram, Hisham A. Al eissa, Murtadha A. Alhumaid, Ali N. Bukhamseen, Koblan M. Al mutared, Abdullah H. Aljwisim, Abdullah M. Twibah, Meteab M. AlSaeed, Hussien A. Alkhalaf, Fatemah M. ALShakhs, Thoyaja Koritala, Jaffar A. Al-Tawfiq, Kuldeep Dhama, Ali A. Rabaan, Awad Al-Omari

**Affiliations:** 1 Administration of Pharmaceutical Care, Al-Ahsa Health Cluster, Ministry of Health, Rashdiah Street, P. O. Box 12944, Alahsa, 31982 Saudi Arabia; 2 Research Center, Almoosa Specialist Hospital, Al-Ahsa, Saudi Arabia; 3 College of Nursing, Princess Norah Bint Abdul Rahman University, Riyadh, Saudi Arabia; 4 School of Nursing, University of Wollongong, Wollongong, Australia; 5 Gastroenterology Department, King Fahad Hofuf Hospital, Ministry of Health, Al-Ahsa, Saudi Arabia; 6 General Surgery Department, Alomran General Hospital, Alahsa, Saudi Arabia; 7 Department of Pharmacy, Hereditary Blood Diseases Centre, Al-Ahsa, Saudi Arabia; 8 Infection Prevention and Control Department, Alomran General Hospital, Alahsa, Saudi Arabia; 9 Pharmaceutical Care Department, Prince Sultan Cardiac Centre, Al-Ahsa, Saudi Arabia; 10 Pharmacy Department, Aljafr General Hospital, Al-Ahsa, Saudi Arabia; 11 Intensive Care Unit, Omran General Hospital, Al-Ahsa, Saudi Arabia; 12 Pharmacy Department, Prince Saud Bin Jalawi Hospital, Al-Ahsa, Saudi Arabia; 13 Pharmacy Department, King Faisal General Hospital, Al-Ahsa, Saudi Arabia; 14 Department of Gastroenterology, King Fahad Hofuf Hospital, Ministry of Health, Al-Ahsa, Saudi Arabia; 15 Critical Care Medicine/Gastroenterology Department, King Fahad Hofuf Hospital, Ministry of Health, Al-Ahsa, Saudi Arabia; 16 Department of Critical Care, King Fahad Hofuf Hospital, Ministry of Health, Al-Ahsa, Saudi Arabia; 17 Administration of Academic Affairs and Research, Ministry of Health, Al-Ahsa, Saudi Arabia; 18 College of Medicine, King Khalid University Hospital, Riyadh, Saudi Arabia; 19 Medical Services Department, King Fahad Hofuf Hospital, Al-Ahsa, Saudi Arabia; 20 Pharmacy Department, Aloyoon General Hospital, Al-Ahsa, Saudi Arabia; 21 Pharmacy Department, Maternity and Children Hospital, Al-Ahsa, Saudi Arabia; 22 Administration of Pharmaceutical Care, Ministry of Health, Najran, Saudi Arabia; 23 Administration of Compliance, Al-Ahsa Health Affairs, Ministry of Health, Al‑Ahsa, Saudi Arabia; 24 Regional Medical Supply, Al-Ahsa Health Cluster, Ministry of Health, Al-Ahsa, Saudi Arabia; 25 Pharmacy Department, Al Jaber Hospital for Eye, Ear, Nose and Throat, Ministry of Health, Al-Ahsa, Saudi Arabia; 26 Respiratory Therapy Department, Prince Saud Bin Jalawi Hospital, Ministry of Health, Al-Ahsa, Saudi Arabia; 27 Department of Internal Medicine, Mayo Clinic Health System, Mankato, MN USA; 28 Infectious Disease Unit, Specialty Internal Medicine, Johns Hopkins Aramco Healthcare, Dhahran, Saudi Arabia; 29 Infectious Disease Division, Department of Medicine, Indiana University School of Medicine, Indianapolis, IN USA; 30 Infectious Disease Division, Department of Medicine, Johns Hopkins University School of Medicine, Baltimore, MD USA; 31 Division of Pathology, ICAR-Indian Veterinary Research Institute, Uttar Pradesh Izatnagar, Bareilly, 243122 India; 32 Molecular Diagnostic Laboratory, Johns Hopkins Aramco Healthcare, Dhahran, Saudi Arabia; 33 College of Medicine, Alfaisal University, Riyadh, 11533 Saudi Arabia; 34 Department of Public Health and Nutrition, The University of Haripur, Haripur, 22610 Pakistan; 35 College of Medicine, Alfaisal University, Riyadh, Saudi Arabia; 36 Research Center, Dr. Sulaiman Al Habib Medical Group, Riyadh, Saudi Arabia

**Keywords:** SARS-Cov-2, Cancer, Colon, Colorectal, COVID-19, Rectum, Meta-analysis, Systematic review

## Abstract

**Background:**

Patients with colorectal cancer (CRC) are more likely to develop severe course of severe acute respiratory syndrome coronavirus 2 (SARS-CoV-2) infection and experience increased risk of mortality compared to SARS-CoV-2 patients without CRC.

**Objectives:**

To estimate the prevalence of SARS-CoV-2 infection in CRC patients and analyse the demographic parameters, clinical characteristics and treatment outcomes in CRC patients with COVID-19 illness.

**Methods:**

For this systematic review and meta-analysis, we searched Proquest, Medline, Embase, Pubmed, CINAHL, Wiley online library, Scopus and Nature for studies on the incidence of SARS-CoV-2 infection in CRC patients, published from December 1, 2019 to December 31, 2021, with English language restriction. Effect sizes of prevalence were pooled with 95% confidence intervals (CIs). Sub-group analyses were performed to minimize heterogeneity. Binary logistic regression model was used to explore the effect of various demographic and clinical characteristics on patient’s final treatment outcome (survival or death).

**Results:**

Of the 472 papers that were identified, 69 articles were included in the systematic review and meta-analysis (41 cohort, 16 case-report, 9 case-series, 2 cross-sectional, and 1 case-control studies). Studies involving 3362 CRC patients with confirmed SARS-CoV-2 (all patients were adults) were analyzed. The overall pooled proportions of CRC patients who had laboratory-confirmed community-acquired and hospital-acquired SARS-CoV-2 infections were 8.1% (95% CI 6.1 to 10.1, *n* = 1308, 24 studies, *I*^*2*^ 98%, *p* = 0.66), and 1.5% (95% CI 1.1 to 1.9, *n* = 472, 27 studies, *I*^*2*^ 94%, *p* < 0.01). The median patient age ranged from 51.6 years to 80 years across studies. The majority of the patients were male (*n* = 2243, 66.7%) and belonged to White (Caucasian) (*n* = 262, 7.8%), Hispanic (*n* = 156, 4.6%) and Asian (*n* = 153, 4.4%) ethnicity. The main source of SARS-CoV-2 infection in CRC patients was community-acquired (*n* = 2882, 85.7%; *p* = 0.014). Most of those SARS-CoV-2 patients had stage III CRC (*n* = 725, 21.6%; *p* = 0.036) and were treated mainly with surgical resections (n = 304, 9%) and chemotherapies (*n* = 187, 5.6%), *p* = 0.008. The odd ratios of death were significantly high in patients with old age (≥ 60 years) (OR 1.96, 95% CI 0.94–0.96; *p* < 0.001), male gender (OR 1.44, 95% CI 0.41–0.47; *p* < 0.001) CRC stage III (OR 1.54, 95% CI 0.02–1.05; *p* = 0.041), CRC stage IV (OR 1.69, 95% CI 0.17–1.2; *p* = 0.009), recent active treatment with chemotherapies (OR 1.35, 95% CI 0.5–0.66; *p* = 0.023) or surgical resections (OR 1.4, 95% CI 0.8–0.73; *p* = 0.016) and admission to ICU (OR 1.88, 95% CI 0.85–1.12; *p* < 0.001) compared to those who survived.

**Conclusion:**

SARS-CoV-2 infection in CRC patient is not uncommon and results in a mortality rate of 26.2%. Key determinants that lead to increased mortality in CRC patients infected with COVID-19 include older age (≥ 60 years old); male gender; Asian and Hispanic ethnicity; if SARS-CoV-2 was acquired from hospital source; advanced CRC (stage III and IV); if patient received chemotherapies or surgical treatment; and if patient was admitted to ICU, ventilated or experienced ARDS.

## Background

Since its outbreak in China in December 2019, corona virus disease 2019 (COVID-19) has spread across the world to become a global pandemic. According to the World Health Organization (WHO), as of July 21, 2022, 562,672,324 confirmed cases of COVID-19 have been recorded worldwide, with 6,367,793 deaths [1]. Established, probable, and possible comorbidities that have been associated with severe COVID-19 in at least 1 meta-analysis or systematic review, in observational studies, or in case series were: age ≥ 65 years, asthma, cancer, cerebrovascular disease, chronic kidney disease, chronic lung disease (interstitial lung disease, pulmonary embolism, pulmonary hypertension, bronchiectasis, chronic obstructive pulmonary disease), chronic liver disease (cirrhosis, non-alcoholic fatty liver disease, alcoholic liver disease, autoimmune hepatitis), diabetes mellitus, type 1 and type 2, heart conditions (such as heart failure, coronary artery disease, or cardiomyopathies), human immunodeficiency virus (HIV), obesity (BMI ≥ 30 kg/m^2^) and overweight (BMI 25 to 29 kg/m^2^), pregnancy or recent pregnancy, primary immunodeficiencies, smoking (current and former), sickle cell disease or thalassemia, solid organ or blood stem cell transplantation, tuberculosis, use of corticosteroids or other immunosuppressive medications [2–4]. In a systematic analysis that calculated the total number of community infections through seroprevalence surveys from 53 countries prior to vaccine availability, the COVID-19 infection mortality rate was 0.005 percent at 1 year, decreased to 0.002 percent by age 7, and increased exponentially after that: 0.006 percent at age 15, 0.06 percent at age 30, 0.4 percent at age 50, 2.9 percent at age 70, and 20 percent at age 90 [5].

**Fig. 1:**
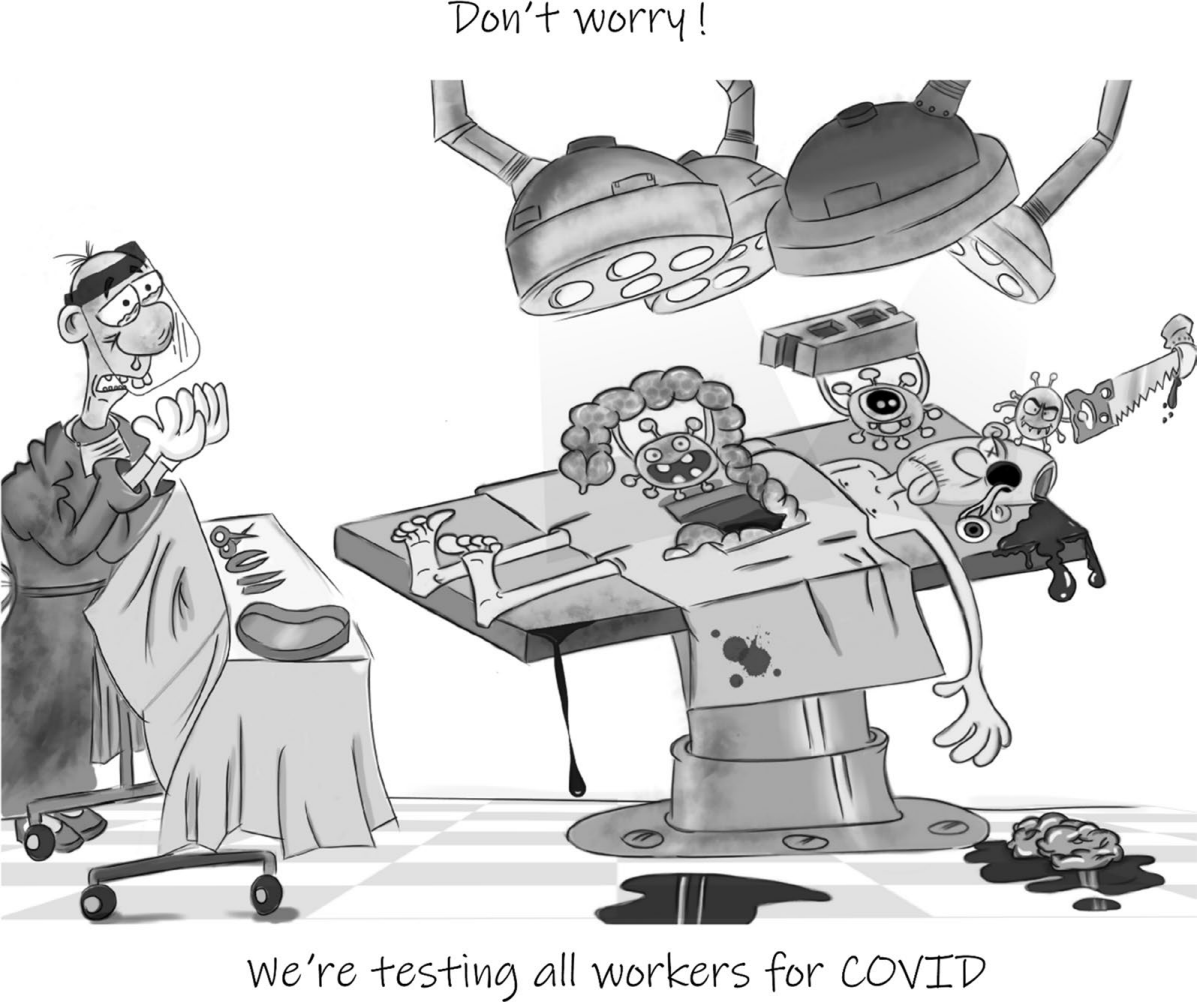
A caricature depicts surgeon’s worriment about contracting the SARS-CoV-2 and patient’s possible risk of getting post-surgical SARS-CoV-2 infection in a CRC patient

Colorectal cancer (CRC) is common and deadly disease, and globally, CRC still remains the third most commonly diagnosed cancer in males and the second in females [6]. CRC is the most common gastrointestinal malignancy and disproportionately affects medically underserved populations [7]. Patients with CRC are more likely to develop severe course of severe acute respiratorcy syndrome coronavirus 2 (SARS-CoV-2) infection and experience increased risk of mortality compared to SARS-CoV-2 patients without CRC [8–16]. Higher mortality rates in CRC patients infected with COVID-19 case-series and cohort studies were reported; for instance, in two small Chinese case-series, rates of death reached up to 61.5 to 70% [11, 15], and in a large French cohort (a total of 376 CRC patients infected with COVID-19 cases), mortality rate was 37.8% [8]; and there was a lower proportion of death of all hospitalized CRC patients infected with COVID-19 based on two different studies in China (5.9%) and Turkey (6.4%) [14, 17]. The recent UK Coronavirus Cancer Monitoring Project (UKCCMP) prospective cohort study of 2,515 patients conducted at 69 UK cancer hospitals among adult patients (≥ 18 years) with an active cancer and COVID-19 reported a 38% (966 patients) mortality rate with an association between higher mortality in patients with haematological malignant neoplasms, particularly in those with acute leukaemias or myelodysplastic syndrome (OR, 2.16; 95% CI, 1.30–3.60) and myeloma or plasmacytoma (OR, 1.53; 95% CI, 1.04–2.26) [18]. Lung cancer was also significantly associated with higher COVID-19-related mortality (OR, 1.58; 95% CI, 1.11–2.25) [18]. A possible reason for increased mortality due to SARS-CoV-2 in patients with CRC is because most health care systems have been required to reorganize their infrastructure and staffing to manage the COVID-19 pandemic [19]. The pandemic has called for a review of healthcare workers daily medical practices, including our approach to CRC management where treatment puts patients at high risk of virus exposure. Given their higher median age, CRC patients are at an increased risk for severe symptoms and complications in cases of infection, especially in the setting of immunosuppression. Considering that the reduction in CRC screening following SARS-CoV-2 pandemic is due to the restrictions imposed for the high prevalence of COVID-19 illness and the lack of referrals due to the fear of developing SARS-CoV-2 infection [20–22] (see Fig. 1).

To date, some studies have been performed to evaluate the SARS-CoV-2 infection in CRC patients, but the results of these studies were inconsistent because most of these are single-centre studies with limited sample sizes [23–36]. In light of newer case reports, case-series and cohort studies that were done to re-evaluate the development of COVID-19 disease in CRC patients, we aimed to estimate the prevalence of SARS-CoV-2 infection in CRC patients and analyse the demographic parameters, clinical characteristics and treatment outcomes in CRC patients with COVID-19 illness with larger and better-quality data.

## Methods

### Design

We followed the Preferred Reporting Items for Systematic Reviews and Meta-Analyses guidelines (PRISMA) in conducting this systematic review and meta-analysis [37]. The following electronic databases were searched: PROQUEST, MEDLINE, EMBASE, PUBMED, CINAHL, WILEY ONLINE LIBRARY, SCOPUS and NATURE with Full Text. We used the following keywords: *COVID-19* OR *SARS-CoV-2* OR *Severe acute Respiratory Syndrome Coronavirus 2* OR *Coronavirus Disease 2019* OR *2019 novel coronavirus* AND *colorectal cancer* OR *colon* OR *rectal* OR *rectum* OR *CRC* OR *bowel cancer* OR *tumor* OR *cancer* OR *neoplasm*. The search was limited to papers published in English between 1 December 2019 and 31 December 2021. Based on the title and abstract of each selected article, we selected those discussing and reporting occurrence of CRC in COVID-19 patients.

### Inclusion–exclusion criteria

Inclusion criteria are as follows: (1) published case reports, case series and cohort studies that focused on COVID-19 in CRC patients that included children and adults as our population of interest; (2) studies of experimental or observational design reporting the prevalence of SARS-CoV-2 infection in patients with CRC; (3) the language was restricted to English.

The exclusion criteria are as follows: (1) editorials, commentaries, case and animal studies, discussion papers, preprints, news analyses, reviews and meta-analyses; (2) studies that did not report data on CRC and SARS-CoV-2; (3) studies that never reported details on SARS-CoV-2 identified cases with CRC; (4) studies that reported CRC in patients with negative COIVD-19 PCR tests; (5) duplicate publications.

### Data extraction

Six authors critically reviewed all of the studies retrieved and selected those judged to be the most relevant. Data were carefully extracted from the relevant research studies independently. Articles were categorized as case report, case series, cross-sectional, case–control or cohort studies. The following data were extracted from selected studies: authors; publication year; study location; study design and setting; age; proportion of male patients; patient ethnicity; methods used for CRC diagnosis; total number of patients and number of CRC patients with positive PCR SARS-CoV-2; source of SARS-CoV-2 infection [community-acquired or hospital-acquired]; CRC staging; treatments received; symptoms from tumor; comorbidities; if patient was admitted to intensive care unit (ICU), placed on mechanical ventilation, and/or suffered acute respiratory distress syndrome (ARDS); assessment of study risk of bias; and treatment outcome (survived or died); which are noted in Table 1.

**Table 1:** Summary of the characteristics of the included studies with evidence on colorectal cancer and SARS-CoV-2 (*n* = 69 studies), 2020–2021

Author, year, study location	Study design, setting	Age (years)^a^	Male, n (%)	Ethnicity^b^	CRC diagnosed through	Number of patients (*n* = 98,131)	Number of SARS-CoV-2 patients with CRC (%) [*n* = 3362, 3.43%]	Source of SARS-CoV-2 infection	CRC Staging	Treatment, n	Symptoms from the tumor, n	Comorbidities, n	Admitted to ICU, n	Mechanical ventilation, n	ARDS, n	NOS score; and Treatment outcome
Al-Shamsi et al. 2020 [23], United Arab Emirates	Prospective, cohort, single centre	51.6 (40-76)	1 (50)	2 Arabs	Symptoms, endoscopy, radiological imaging, biopsies and tumor markers	85	2	Community-acquired	Not reported	2 Chemotherapies	Not reported	Not reported	1	0	0	(NOS, 7) 2 survived
Aschele et al. 2021 [43], Italy	Retrospective, cohort, multicentre	68 (28–89)	Not reported	38 Whites, (Caucasians)	Symptoms, endoscopy, radiological imaging, biopsies and tumor markers	406	38	Community-acquired	Not reported	28 Chemotherapies 16 Monoclonal antibodies: [CRC use (*n* = 5) and COVID-19 use (*n* = 11)] Immunotherapies 0 Targeted therapies 3 Hormones	Not reported	Not reported	Not reported	Not reported	Not reported	(NOS, 7) Treatment outcome was not available
Ayhan et al. 2021 [44], Turkey	Retrospective, cohort, single centre	61.0 (21–84)	Not available	11 Whites (Caucasians)	Symptoms, endoscopy, radiological imaging, biopsies and tumor markers	84	11	Community-acquired	Stage I (*n* = 1) Stage II (*n* = 3) Stage III (*n* = 1) Stage IV (*n* = 6)	8 Chemotherapies 5 Targeted therapies 2 Immunotherapies	Not reported	3 Hypertension 5 Diabetes mellitus 2 Coronary artery disease 1 COPD 1 Chronic renal failure	Not reported	Not reported	Not reported	(NOS, 6) Treatment outcome was not available
Aznab 2020 [45], Iran	Retrospective, cohort, single centre	Not reported	Not reported	72 Persians	Not reported	279	72	Community-acquired	Stage II (*n* = 11) Stage III (*n* = 36) Stage IV (*n* = 25)	72 Chemotherapies 25 Monoclonal antibodies: [CRC use (*n* = 9) and COVID-19 use (*n* = 16)]	Not reported	Not reported	Not reported	Not reported	1	(NOS, 6) 71 survived 1 died
Berger et al. 2021 (25), Austria	Retrospective, cohort, single centre	Not reported	1 (100)	1 White (Caucasian)	Symptoms, endoscopy, radiological imaging, biopsies and tumor markers	23	1	Hospital- acquired	Not reported	1 Chemotherapy 1 Targeted therapy 1 Immunotherapy	Not reported	No comorbidities	0	0	0	(NOS, 7) 1 survived
Bernard et al. 2021 [8], France	Retrospective, cohort, multicentre	73 (64–82)	Not reported	Multi-ethnic	Symptoms, endoscopy, radiological imaging, biopsies and tumor markers	6201	518	Community-acquired	Not reported	Not reported	Not reported	Not possible to extract	67	Not possible to extract	Not possible to extract	(NOS, 8) 376 survived 142 died
Binet et al. 2021 [46], Belgium	Retrospective, case report, single centre	62	0 (0)	1 White (Caucasian)	Symptoms, endoscopy, radiological imaging, biopsies and tumor markers	1	1	Hospital- acquired	Stage IV (*n* = 1)	1 Colectomy	1 Abdominal pain 1 Nausea and vomiting	1 Hypertension 1 Diabetes mellitus 1 Dyslipidaemia 1 Acute ischemic stroke	0	0	0	(NOS, 6) 1 died
Calvo et al. 2021 [47], Spain	Retrospective, case-series, single centre	63.9 ± (10.2)	3 (60)	5 Whites (Caucasians)	Symptoms, endoscopy, radiological imaging, biopsies and tumor markers	653	5	Hospital- acquired	Stage IV (*n* = 3)	3 Chemotherapies 2 Radiotherapies 3 Steroids 3 Antivirals 3 HCQ 3 Surgical resections 1 Immunotherapies	Not reported	Not possible to extract	Not possible to extract	Not possible to extract	Not possible to extract	(NOS, 6) 3 survived 2 died
COVIDSurg Collaborative 2021 [9], 40 countries	Prospective, cohort, multicentre	< 50: *n* = 174; 50–69: *n* = 966; AND ≥ 70: *n* = 933	1236 (59.6)	Multi-ethnic	Symptoms, endoscopy, radiological imaging, biopsies and tumor markers	2073	78	Hospital- acquired	Stage I-II (*n* = 838) Stage III (*n* = 653) Stage IV (*n* = 133)	38 Surgical resections	Not reported	Not possible to extract	Not possible to extract	Not possible to extract	Not possible to extract	(NOS, 7) 63 survived 15 died
Cosma et al. 2020 [48], Italy	Retrospective, case report, single centre	Not reported	Not reported	1 White (Caucasian)	Symptoms, endoscopy, radiological imaging, biopsies and tumor markers	1	1	Community-acquired	Stage IV (*n* = 1)	Not reported	Not reported	No comorbidities	0	0	0	(NOS, 5) 1 survived
Costanzi et al. 2020 [49], Italy	Retrospective, case report, single centre	62	0 (0)	1 White (Caucasian)	Symptoms, endoscopy, radiological imaging, biopsies and tumor markers	1	1	Hospital- acquired	Stage IV (*n* = 1)	1 Surgical resection 1 Chemotherapy 1 Ileostomy 1 Antivirals 1 Antibiotics 1 HCQ 1 RBC transfusion	1 Haematochezia (blood per anus) 1 Anaemia (unexplained iron deficiency) 1 Blood per rectum 1 Weight loss	No comorbidities	0	0	0	(NOS, 6) 1 survived
Filipe et al. 2021 [26], The Netherlands	Retrospective, cohort, multicentre	-	-	2 Whites (Caucasians)	Symptoms, endoscopy, radiological imaging, biopsies and tumor markers	21	2	Hospital- acquired	Stage II (*n* = 1) Stage IV (*n* = 1)	2 Chemotherapies 2 Surgical resections	Not reported	Not reported	Not reported	Not reported	Not reported	(NOS, 7) 1 survived 1 died
Gao et al. 2020 [50], China	Retrospective, case report, single centre	69	0 (0)	1 Asian	Symptoms and exploratory laparotomy	1	1	Community-acquired	Not reported	1 Colectomy 1 Lymph node dissection	1 Abdominal pain 1 Change in bowel habits 1 Weight loss	No comorbidities	0	0	0	(NOS, 7) 1 survived
Glasbey et al. 2021 [51], 55 countries	Prospective, cohort, multicentre	-	-	Multi-ethnic	Symptoms, endoscopy, radiological imaging, biopsies and tumor markers	2310	134	Hospital- acquired	Not reported	134 Surgical resections	Not reported	Not possible to extract	Not possible to extract	Not possible to extract	Not possible to extract	(NOS, 7) Treatment outcome was not available
Haque et al. 2021 [52], United Kingdom	Retrospective, case report, single centre	69	1 (100)	1 White (Caucasian)	Symptoms, CT and colonoscopy	1	1	Community-acquired	Not reported	1 RBC transfusions 1 Antibiotics 1 Tranexamic acid 1 Palliative haemostatic radiotherapy	1 Melena (black tarry stools) 1 Anaemia (unexplained iron deficiency)	1 Lynch Syndrome 1 Pancolectomy 1 Ileo-rectal anastomosis 1 Adenocarcinoma 1 Nephroureterectomy 1 Recurrent VTE	0	0	0	(NOS, 7) 1 survived
Huang et al. 2020 [141], China	Retrospective, case report, single centre	48	1 (100)	1 Asian	Symptoms, and CT	1	1	Hospital- acquired	Stage II (*n* = 1)	1 Sigmoidectomy 1 Colonic decompression 1 Anastomosis 1 Ileostomy	1 Abdominal pain 1 Constipation	1 Hepatitis B virus	0	0	0	(NOS, 6) 1 survived
Joharatnam-Hogan et al. 2020 [53], United Kingdom	Retrospective, cohort, multicentre	76 (72–77.5)	4 (80)	4 Whites (Caucasi ans) 1 Black	Symptoms, endoscopy, radiological imaging, biopsies and tumor markers	699	5	Hospital- acquired	Not reported	2 Chemotherapies 1 Colectomy 2 Surgical resections 1 Radiotherapy	3 Anaemia (unexplained iron deficiency)	Not reported	Not reported	Not reported	Not reported	(NOS, 6) 5 survived
Johnson et al. 2020 [54], United States	Retrospective, case report, single centre	63	1 (100)	1 Asian	Symptoms, endoscopy, radiological imaging, biopsies and tumor markers	1	1	Hospital- acquired	Stage IV (*n* = 1)	1 Chemotherapy 1 Hepatectomy	Not reported	1 Lynch syndrome 1 Colon, liver, and thyroid cancer 1 Hepatitis C virus	0	0	0	(NOS, 6) 1 survived
Karam et al. 2020 [55], Australia	Retrospective, case report, single centre	66	1 (100)	1 White (Caucasian)	Symptoms, and CT	1	1	Community-acquired	Not reported	1 Antibiotics 1 Surgical debridement 1 Colostomy	1 Haematochezia (blood per anus) 1 Anaemia (unexplained iron deficiency) 1 Change in bowel habits	1 Diabetes mellitus 1 Renal impairment 1 Chronic anaemia 1 Diabetic ketoacidosis	0	0	0	(NOS, 6) 1 survived
Khan et al. 2021 [27], United Kingdom	Retrospective, case-series, single centre	78	1 (100)	1 White (Caucasian)	Symptoms, endoscopy, radiological imaging, biopsies and tumor markers	8	1	Hospital- acquired	Stage IV (*n* = 1)	1 Conservative treatment	1 Abdominal pain 1 Change ion bowel habits	1 Heart failure 1 Chronic kidney disease	1	1	1	(NOS, 6) 1 died
Kuryba et al. 2021 [56], United Kingdom	Retrospective, cohort, multicentre	60–74: 43.05%; AND 50–69: 26.3%	54 (55.6)	Multi-ethnic	Symptoms, endoscopy, radiological imaging, biopsies and tumor markers	3227	97	Hospital- acquired	Stage I (*n* = 30) And Stage ≥ II (*n* = 23)	83 Surgical resections 33 Colectomies 6 Hartmann’s procedure 5 Stomas 2 Stents	Not reported	Not reported	Not reported	Not reported	Not reported	(NOS, 6) Treatment outcome was not available
Kumar et al. 2020 [68], India	Ambispective, cohort, single centre	Not reported	Not reported	10 Indians	Symptoms, endoscopy, radiological imaging, biopsies and tumor markers	107	10	Hospital- acquired	Not reported	4 Radiotherapies 8 Chemotherapies 10 Surgeries 2 Palliative managements 1 Stoma closure	Not reported	Not reported	Not reported	Not reported	Not reported	(NOS, 6) 10 survived
Larfors et al. 2021 [10], Sweden	Retrospective, cohort, multicentre	Not reported	Not reported	Multi-ethnic	Symptoms, endoscopy, radiological imaging, biopsies and tumor markers	54,651	50	Hospital- acquired	Not reported	22 Chemotherapies	Not reported	Not reported	50	Not reported	Not reported	(NOS, 6) 50 died
Liang et al. 2020 [57], China	Prospective, cohort, multicentre	67.5 (53.7–85)	4 (100)	4 Asians	Not reported	1590	4	Hospital- acquired	Not reported	3 Surgical resections 3 Chemotherapies	Not reported	1 Diabetes mellitus 1 Hypertension 1 COPD	2	2	Not reported	(NOS, 7) 2 survived 2 died
Liu et al. 2020 [28], China	Retrospective, case-series, single centre	65.5 (54.5–73.0)	3 (60)	6 Asians	Symptoms, endoscopy, radiological imaging, biopsies and tumor markers	52	5	Hospital- acquired	Not reported	5 Surgical resections 2 Sigmoidectomies 1 Colectomy 1 Hartmann's procedure	1 Abdominal pain 2 Diarrhoea	3 Hypertension 1 Diabetes mellitus 1 Tuberculosis	1	1	1	(NOS, 6) 4 survived 1 died
Liu et al. 2021 [58], China	Retrospective, cohort, single centre	All patients were > 60	3 (60)	5 Asians	Symptoms, endoscopy, radiological imaging, biopsies and tumor markers	189	5	Community-acquired	Not reported	5 Surgical resections 2 Chemotherapies 1 Targeted therapy	1 Abdominal pain 3 Change in bowel habits 1 Nausea and vomiting 2 Diarrhoea	2 Hypertension 2 Diabetes 1 Coronary heart disease	1	1	1	(NOS, 6) 4 survived 1 died
Liu et al. 2021 [17], China	Retrospective, cohort, multicentre	65.06 ± (11.51)	23 (63.9)	36 Asians	Symptoms, endoscopy, radiological imaging, biopsies and tumor markers	81	36	Community-acquired	Stage I (*n* = 5) Stage II (*n* = 7) Stage III (*n* = 11) Stage IV (*n* = 7)	21 Conservative treatment 4 Chemotherapies 4 Surgeries 32 Antivirals 28 Antibiotics 16 Steroids 10 IgG	7 Change in bowel habits 9 Diarrhoea	9 Diabetes mellitus 18 Hypertension 3 Cardiovascular disease 4 Cerebrovascular disease 3 COPD	6	5	8	(NOS, 6) 34 survived 2 died
Liu et al. 2021 [58], China	Retrospective, case-series, single centre	> 65: n = 3; AND ≤ 65: n = 2	3 (60)	5 Asians	Symptoms, endoscopy, radiological imaging, biopsies and tumor markers	135	5	Community-acquired	Stage I (*n* = 2); Stage II (*n* = 2); and Stage III (*n* = 1)	4 Antibiotics 3 Antivirals 3 Immunomodulator 4 Systemic glucocorticoids	Not reported	1 Diabetes mellitus 3 Hypertension 1 Coronary heart disease 1 Renal disease	1	1	1	(NOS, 8) 4 survived 1 died
Ma et al. 2020 [11], China	Retrospective, cohort, single centre	62 (59–70)	6 (54.5)	11 Asians	Not available	1380	11	Community-acquired	Not reported	4 Surgeries 3 Radiotherapies 7 Chemotherapies 2 Targeted therapies 2 Immunotherapies	2 Haematochezia (blood per anus) 3 Diarrhoea	3 Diabetes mellitus 2 Hypertension 1 COPD	4	4	4	(NOS, 7) 7 survived 4 died
Manlubatan et al. 2021 [59], Philippines	Retrospective, case report, single centre	75	1 (100)	1 Asian	Symptoms, and CT	1	1	Community-acquired	Stage IV (*n* = 1)	1 Abdominotransanal resection 1 Total mesorectal excision 1 Intersphincteric resection 1 Stoma was created	1 Haematochezia (blood per anus) 1 Melena (black tarry stools) 1 Change in bowel habits 1 Blood per rectum 1 Weight loss	No comorbidities	0	0	0	(NOS, 6) 1 died
Mansi et al. 2021 [29], France	Prospective, cohort, multicentre	70.5 (69–70.5)	1 (50)	2 Whites (Caucasians)	Symptoms, endoscopy, radiological imaging, biopsies and tumor markers	28	2	Community-acquired	Stage III (*n* = 1) Stage IV (*n* = 1)	2 Chemotherapies 1 Monoclonal antibody: [CRC use (*n* = 1)]	Not reported	1 Dyslipidemia	1	1	1	(NOS, 7) 1 survived 1 died
Martín-Bravo et al. 2021 [60], Spain	Retrospective, cohort, multicentre	64 (52–64)	1 (50)	2 Whites (Caucasians)	Symptoms, endoscopy, radiological imaging, biopsies and tumor markers	673	2	Hospital-acquired	Stage IV (*n* = 2)	2 Chemotherapies 2 Surgical resections 2 Palliative treatments 1 Immunotherapy 1 Targeted therapy 1 Radiotherapy	Not reported	No comorbidities	1	1	1	(NOS, 6) 1 survived 1 died
Martínez et al. 2021 [61], Spain	Retrospective, cohort, single centre	77 (57-80)	2 (66.7)	3 Whites (Caucasians)	Symptoms, endoscopy, radiological imaging, biopsies and tumor markers	32	3	Hospital-acquired	Stage II (*n* = 3)	1 Sigmoidectomy 1 Hartmann procedure 1 Colostomy 1 Laparoscopic approach 1 Stoma creation	1 Melena (black tarry stools) 1 Diarrhoea	1 Diabetes mellitus 1 Hypertension 1 COPD	0	0	0	(NOS, 7) 3 survived
Martínez-Mardones et al. 2021 [30], Chile	Retrospective, case-series, single centre	72	1 (100)	1 Hispanic	Symptoms, endoscopy, radiological imaging, biopsies and tumor markers	16	1	Hospital-acquired	Stage IV (*n* = 1)	Not reported	1 Haematochezia (blood per anus) 1 Weight loss	Not reported	Not reported	Not reported	Not reported	(NOS, 5) 1 survived
McCarthy et al. 2020 [12], 2 countries	Retrospective, cohort, multicentre	80 (71.5–86.5)	-	Multi-ethnic	Symptoms, endoscopy, radiological imaging, biopsies and tumor markers	Not reported	1564	Community-acquired	Not reported	Not reported	Not reported	Not reported	Not reported	Not reported	Not reported	(NOS, 6) 1139 survived 425 died
Mehta et al. 2020 [62], United States	Retrospective, cohort, single centre	50–60: n = 1; 60–70: n = 3; 70–80: n = 1; 80–90: n = 2	6 (85.7)	Multi-ethnic	Not reported	218	21	Community-acquired	Not reported	Not reported	Not reported	2 Morbid obesity 2 Congestive heart failure 2 Hypertension 1 Diabetes mellitus 1 Coronary artery disease 1 Chronic kidney disease 1 Hepatitis C virus 1 COPD 1 Malabsorption	5	5	7	(NOS, 6) 13 survived 8 died
Miyashita et al. 2020 [63], United States	Retrospective, cohort, single centre	Not reported	Not reported	Multi-ethnic	Not reported	334	16	Community-acquired	Not reported	Not reported	Not reported	Not possible to extract	Not possible to extract	Not possible to extract	Not possible to extract	(NOS, 7) Treatment outcome was not available
Montopoli et al. 2020 [64], Italy	Retrospective, cohort, multicentre	Not reported	Not reported	Multi-ethnic	Not reported	9280	65	Community-acquired	Not reported	Not reported	Not reported	Not possible to extract	Not possible to extract	Not possible to extract	Not possible to extract	(NOS, 7) Treatment outcome was not available
Nagarkar et al. 2021 [65], India	Retrospective, cohort, single centre	Not reported	Not reported	53 Indians	Symptoms, endoscopy, radiological imaging, biopsies and tumor markers	458	53	46 Community-acquired 7 Hospital-acquired	Not reported	Not reported	Not reported	Not possible to extract	Not possible to extract	Not possible to extract	Not possible to extract	(NOS, 6) Treatment outcome was not available
Nakamura et al. 2021 [31], Japan	Retrospective, cohort, single centre	70.5 (54–70.5)	1 (50)	2 Asian	Symptoms, endoscopy, radiological imaging, biopsies and tumor markers	32	2	Hospital-acquired	Stage IV (*n* = 1)	2 Antivirals 2 Steroids 2 Chemotherapies 2 Surgical resections	Not reported	2 Hypertension 1 Coronary heart disease 1 Diabetes mellitus 1 Asthma	1	1	1	(NOS, 6) 1 survived 1 died
Ospina et al. 2021 [13], Colombia	Ambispective, cohort, multicentre	50–60: 23.34%; 61–70: 22.24%; AND > 70: 27.22%	Not reported	92 Hispanics	Symptoms, endoscopy, radiological imaging, biopsies and tumor markers	742	92	Community-acquired	Stage I (*n* = 37) Stage II (*n* = 21) Stage III (*n* = 10) Stage IV (*n* = 24)	Not possible to extract	Not reported	Not reported	Not reported	19	Not reported	(NOS, 6) 64 survived 28 died
Ottaiano et al. 2021 [32], Italy	Retrospective, case reports, single centre	60 (58–60)	2 (66.7)	3 Whites (Caucasians)	Symptoms, endoscopy, radiological imaging, biopsies and tumor markers	3	3	Hospital-acquired	Stage III (*n* = 2) Stage IV (*n* = 1)	3 Chemotherapies 3 Colectomies 1 Monoclonal antibody: [CRC use (*n* = 1)]	Not reported	1 Peritoneal disease 1 Lung disease	0	0	0	(NOS, 6) 3 survived
Özdemir et al. 2021 [14], Turkey	Ambispective, cohort, multicentre	61 (19–94)	771 (50.6)	165 Whites (Caucasians)	Symptoms, endoscopy, radiological imaging, biopsies and tumor markers	1523	165	Community-acquired	Not reported	Not possible to extract	Not reported	Not possible to extract	Not possible to extract	Not possible to extract	Not possible to extract	(NOS, 6) 155 survived 10 died
Pawar et al. 2020 [66], India	Retrospective, case report, single centre	28	0 (0)	1 Indian	Symptoms, endoscopy, radiological imaging, biopsies and tumor markers	1	1	Community-acquired	Not reported	1 Laparoscopic anterior resection	1 Blood per rectum	No comorbidities	0	0	0	(NOS, 5) 1 survived
Pertile et al. 2021 [33], Italy	Retrospective, case-series, single centre	76	1 (100)	1 White (Caucasian)	Symptoms, endoscopy, radiological imaging, biopsies and tumor markers	25	1	Community-acquired	Stage IV (*n* = 1)	1 RBC transfusions 1 Laparotomy 1 Rectal resection 1 Radical cystectomy 1 Right ureterostomy 1 Segmental small bowel resection 1 Lumbar-aortic lymphadenectomy 1 HCQ 1 Antibiotics	1 Anaemia (unexplained iron deficiency) 1 Blood per rectum	1 Chronic renal failure 1 Bilateral ureteral stenting 1 Hypertension 1 Paroxysmal atrial fibrillation	1	1	1	(NOS, 6) 1 died
Pordány et al. 2020 [67], Hungary	Retrospective, case report, single centre	75	0 (0)	1 White (Caucasian)	Symptoms, endoscopy, radiological imaging, biopsies and tumor markers	1	1	Community-acquired	Stage IV (*n* = 1)	1 Colectomy	Not reported	1 Cardiac arrest	1	1	1	(NOS, 5) 1 survived
Quaquarini et al. 2020 [34], Italy	Retrospective, cohort, single centre	60	1 (100)	1 White (Caucasian)	Symptoms, endoscopy, radiological imaging, biopsies and tumor markers	7	1	Community-acquired	Stage IV (*n* = 1)	1 Chemotherapy	Not reported	1 Hypertension 1 Cardiac failure 1 Renal failure 1 Rheumatoid arthritis	Not reported	Not reported	Not reported	(NOS, 7) Treatment outcome was not available
Robilotti et al. 2020 [69], United States	Retrospective, cohort, single centre	Most patients were adults over the age of 60 years	Not reported	Not reported	Symptoms, endoscopy, radiological imaging, biopsies and tumor markers	2035	37	Hospital-acquired	Not reported	Not possible to extract	Not reported	Not possible to extract	Not possible to extract	Not possible to extract	Not reported	(NOS, 8) Treatment outcome was not available
Ruiz-Garcia et al. 2021 [70], Mexico	Prospective, cohort, multicenter	Not reported	Not reported	56 Hispanics	Symptoms, endoscopy, radiological imaging, biopsies and tumor markers	599	56	Community-acquired	Not reported	Not possible to extract	16 Abdominal pain 11 Nausea and vomiting	Not possible to extract	Not reported	Not possible to extract	Not reported	(NOS, 6) Treatment outcome was not available
Serrano et al. 2020 [71], Spain	Retrospective, case report, single centre	78	1 (100)	1 White (Caucasian)	Symptoms, endoscopy, radiological imaging, biopsies and tumor markers	1	1	Community-acquired	Not reported	1 Antivirals 1 HCQ 1 Interferon beta-1b	Not reported	1 Hypertension 1 Chronic kidney disease	0	0	0	(NOS, 6) 1 survived
Sobrado et al. 2021 [72], Brazil	Retrospective, cross-sectional, single centre	72 (67–72)	3 (60)	5 Hispanics	Symptoms, endoscopy, radiological imaging, biopsies and tumor markers	103	5	Hospital-acquired	Stage III (*n* = 2) Stage IV (*n* = 3)	4 Surgical resections 2 Colectomies 1 Adrenalectomy 1 Colostomy closure 1 Hartmann’s procedure reversal	1 Abdominal pain 1 Change in bowel habits	2 Diabetes mellitus 1 Hypertension 1 Mesenteric ischemia 1 Pulmonary embolism	3	3	3	(NOS, 6) 2 survived 3 died
Sorrentino et al. 2020 [73], Italy	Retrospective, cohort, multicentre	Not reported	Not reported	3 Whites (Caucasians)	Symptoms, endoscopy, radiological imaging, biopsies and tumor markers	Not reported	Not reported	Hospital-acquired	Not reported	Not reported	Not reported	Not reported	Not reported	Not reported	Not reported	(NOS, 6) 2 survived 1 died
Sukumar et al. 2020 [74], India	Prospective, cohort, single centre	Not reported	63 (70)	1 Indian	Symptoms, endoscopy, radiological imaging, biopsies and tumor markers	90	1	Hospital-acquired	Stage II (*n* = 1)	1 Surgical resection 1 Chemotherapy	Not reported	1 Diabetes mellitus 1 Hypertension	0	0	0	(NOS, 6) 1 survived
Tateno et al. 2021 [75], Japan	Retrospective, case report, single centre	63	1 (100)	1 Asian	Symptoms, endoscopy, radiological imaging, biopsies and tumor markers	1	1	Community-acquired	Stage II (*n* = 1)	1 Colectomy 1 Antibiotics 1 Antivirals	Not reported	1 Diabetes mellitus	0	0	0	(NOS, 6) 1 survived
Taya et al. 2021 [76], United States	Retrospective, cross-sectional, single centre	63 (63–68)	3 (30)	7 Whites (Caucasians) 3 Blacks	Symptoms, endoscopy, radiological imaging, biopsies and tumor markers	745	10	Community-acquired	Not reported	Not reported	Not reported	Not reported	Not reported	Not reported	Not reported	(NOS, 6) Treatment outcome was not available
Tejedor et al. 2021 [77], Spain	Prospective, cohort, multicentre	76.5 (69–76.5)	3 (100)	3 Whites (Caucasians)	Symptoms, endoscopy, radiological imaging, biopsies and tumor markers	301	3	Hospital-acquired	Stage II (*n* = 2) Stage IV (n = 1)	1 Chemotherapy 1 Surgical resection 1 Sigmoidectomy 1 Colectomy	Not reported	1 Diabetes mellitus 1 Hypertension	2	0	1	(NOS, 6) 2 survived 1 died
Tolley et al. 2020 [35], United Kingdom	Retrospective, case-series, single centre	67.5 (55–84)	2 (66.7)	3 Whites (Caucasians)	Symptoms, endoscopy, radiological imaging, biopsies and tumor markers	21	3	Hospital-acquired	Stage III (*n* = 2) Stage IV (*n* = 1)	3 Surgeries	Not reported	Not reported	Not reported	Not reported	Not reported	(NOS, 6) 2 survived 1 died
Tolley et al. 2020 [78], United Kingdom	Retrospective, case-series, single centre	Not reported	Not reported	Not reported	Not reported	21	3	Hospital-acquired	Not reported	Not reported	Not reported	Not reported	Not reported	Not reported	Not reported	(NOS, 6) 2 survived 1 died
Tuech et al. 2021 [79], France	Retrospective, cohort, multicentre	Not reported	Not reported	Not reported	Not reported	461	6	Hospital-acquired	Not reported	Not reported	Not reported	3 Hypertension 2 Diabetes mellitus 1 COPD 1 Chronic kidney disease	0	0	0	(NOS, 6) 6 survived
Vicente et al. 2021 [80], Brazil	Retrospective, cohort, single centre	64 (62–64)	1 (50)	2 Hispanics	Symptoms, endoscopy, radiological imaging, biopsies and tumor markers	41	2	Hospital-acquired	Stage II (*n* = 2)	2 Sigmoidectomies 1 Chemotherapy 1 Radiotherapy 1 Ileostomy	Not reported	1 Morbid obesity 1 Diabetes mellitus 1 Hypertension	0	0	0	(NOS, 6) 2 survived
Wang et al. 2021 [81], United States	Retrospective, case–control, multicentre	Not reported	Not reported	Multi-ethnic	Not reported	1200	60	Community-acquired	Not reported	Not reported	Not reported	Not possible to extract	Not reported	Not reported	Not reported	(NOS, 8) Treatment outcome was not available
Wang et al. 2020 [15], China	Retrospective, cohort, multicentre	63 (55–70)	26 (76.5)	34 Asians	Symptoms, endoscopy, radiological imaging, biopsies and tumor markers	283	34	Community-acquired	Most patients were stage I (*n* = 20)	Not possible to extract	11 Change in bowel habits 7 Nausea and vomiting 6 Diarrhoea	Not possible to extract	Not possible to extract	Not possible to extract	Not possible to extract	(NOS, 5) 20 survived 14 died
Woźniak et al. 2021 [82], Poland	Retrospective, case report, single centre	56	1 (100)	1 White (Caucasian)	Symptoms, endoscopy, radiological imaging, biopsies and tumor markers	1	1	Community-acquired	Stage IV (*n* = 1)	1 Chemotherapy 1 Sigmoidectomy	1 Anaemia (unexplained iron deficiency) 1 Weight loss	1 Coronary heart disease 1 Myocardial infarction 1 Coronary artery angioplasty 1 Polyneuropathy	0	0	0	(NOS, 6) 1 survived
Wu et al. 2020 [36], China	Retrospective, case-series, multicenter	29	0 (0)	1 Asian	Symptoms, endoscopy, radiological imaging, biopsies and tumor markers	11	1	Hospital-acquired	Stage IV (*n* = 1)	1 Surgery 1 Immunotherapy 1 Antibiotics 1 Chemotherapy	Not reporter	No comorbidities	1	1	0	(NOS, 6) 1 died
Yang et al. 2020 [16], China	Retrospective, cohort, multicenter	63 (56–70)	Not reported	28 Asians	Symptoms, endoscopy, radiological imaging, biopsies and tumor markers	205	28	Community-acquired	Stage I-II (*n* = 20) Stage III-IV (*n* = 6)	Not possible to extract	Not reported	11 Hypertension 8 Diabetes mellitus 1 Coronary heart disease 1 Hepatitis B virus	Not possible to extract	Not possible to extract	Not possible to extract	(NOS, 8) 22 survived 6 died
Yang et al. 2020 [83], China	Retrospective, cohort, single centre	Not reported	Not reported	11												
Asians	Symptoms, endoscopy, radiological imaging, biopsies and tumor markers	1575	13	Community-acquired	Not reported	13 Antivirals 12 Antibiotics 5 Chemotherapies 3 Steroids 2 Surgical resections 1 Immunotherapy	1 Nausea and vomiting 1 Diarrhoea	8 Hypertension 6 Diabetes mellitus 2 Coronary heart disease 1 COPD 1 Cerebrovascular disease	2	2	2	(NOS, 6) 11 survived 2 died				
Ye et al. 2020 [84], China	Retrospective, case report, single centre	62	1 (100)	1 Asians	Symptoms, endoscopy, and radiological imaging	1	1	Hospital-acquired	Not reported	1 Colectomy 1 Antibiotics 1 Antivirals 1 Antifungals	Not reported	Not reported	0	0	0	(NOS, 5) 1 survived
Yu et al. 2020 [83], China	Retrospective, cohort, single centre	66 (48–78)	2 (100)	2 Asians	Not reported	1524	2	Hospital-acquired	Not reported	1 Best supportive care 1 Newly diagnosed; treatment yet to commence	Not reported	Not reported	0	0	0	(NOS, 7) 2 survived
Zhang et al. 2020 [85], China	Retrospective, cohort, multicentre	77.5 (75–77.5)	2 (100)	2 Asians	Symptoms, endoscopy, radiological imaging, biopsies and tumor markers	1276	2	Hospital-acquired	Not reported	2 Antivirals 2 Antibiotics 1 Surgical operation 1 Chemotherapy 1 Steroids	1 Diarrhoea	1 Hypertension 1 Coronary heart disease 1 COPD	1	1	1	(NOS, 6) 1 survived 1 died

*ARDS* Acute respiratory distress syndrome, *COPD* Chronic obstructive pulmonary disease, *CRC* Colorectal carcinoma, *CT* Computerized tomography, *HCQ* Hydroxychloroquine, *ICU* Intensive care unit, *IgG* Immunoglobulin G, *IV* Intravenous, *NOS* Newcastle ottawa scale, *RBC* Red blood cell, SARS-CoV-2, severe acute respiratory syndrome coronavirus 2, *VTE* Venous thromboembolism

^a^Data are presented as median (25th–75th percentiles), or mean ± (SD)

^b^Patients with black ethnicity include African-American, Black African, African and Afro-Caribbean patients

### Quality assessment

The quality assessment of the studies was undertaken based on the Newcastle–Ottawa Scale (NOS) to assess the quality of the selected studies [38]. This assessment scale has two different tools for evaluating case–control and cohort studies. Each tool measures quality in the three parameters of selection, comparability, and exposure/ outcome, and allocates a maximum of 4, 2, and 3 points, respectively [38]. High-quality studies are scored greater than 7 on this scale, and moderate-quality studies, between 5 and 7 [38]. Quality assessment was performed by six authors independently, with any disagreement to be resolved by consensus.

### Data analysis

We examined primarily the proportion of confirmed SARS-CoV-2 infection in patients with CRC. This proportion was further classified based on source of SARS-CoV-2 infection (if CRC patient contracted SARS-CoV-2 from the community or hospital). Community-acquired SARS-CoV-2 infection is the infection that CRC patients contracted outside the hospital (i.e., SARS-CoV-2 infection that become clinically apparent within 48 h of the hospital admission or CRC patients have had the infection when admitted to the hospital for some other reason) [39]. Hospital-acquired SARS-CoV-2 infection is the infection that CRC patients contracted within the hospital, the SARS-CoV-2 infections contracted within the hospital but not become clinically apparent until after the discharge of the CRC patient, or SARS-CoV-2 infections contracted by the healthcare workers as a result of their direct or indirect contact with the CRC patients [39]. Taking a conservative approach, a random effects with the DerSimoniane-Laird model was used [40], which produces wider confidence intervals (CIs) than a fixed effect model. Results were illustrated using forest plots. The Cochran’s chi-square (*χ*^*2*^) and the *I*^*2*^ statistic provided the tools of examining statistical heterogeneity [41]. An *I*^*2*^ value of > 50% suggested significant heterogeneity [42]. Examining the source of heterogeneity, a subgroup analysis was conducted based on study location (if continent of Asia, America, Europe or multi-countries).

Individual CRC patient data on demographic parameters and clinical variables and associated treatment outcomes (survived or died) were extracted from the included studies. Univariate and multivariable logistic regression analysis were used to estimate odds ratio (OR) and 95% CIs of the association of each variable with the treatment outcomes of CRC patients with SARS-CoV-2 infection. All *p*-values were based on two-sided tests and significance was set at a *p*-value less than 0.05. R version 4.1.0 with the packages *finalfit* and *forestplot* was used for all statistical analyses.

## Results

### Study characteristics and quality

**Fig. 2:**
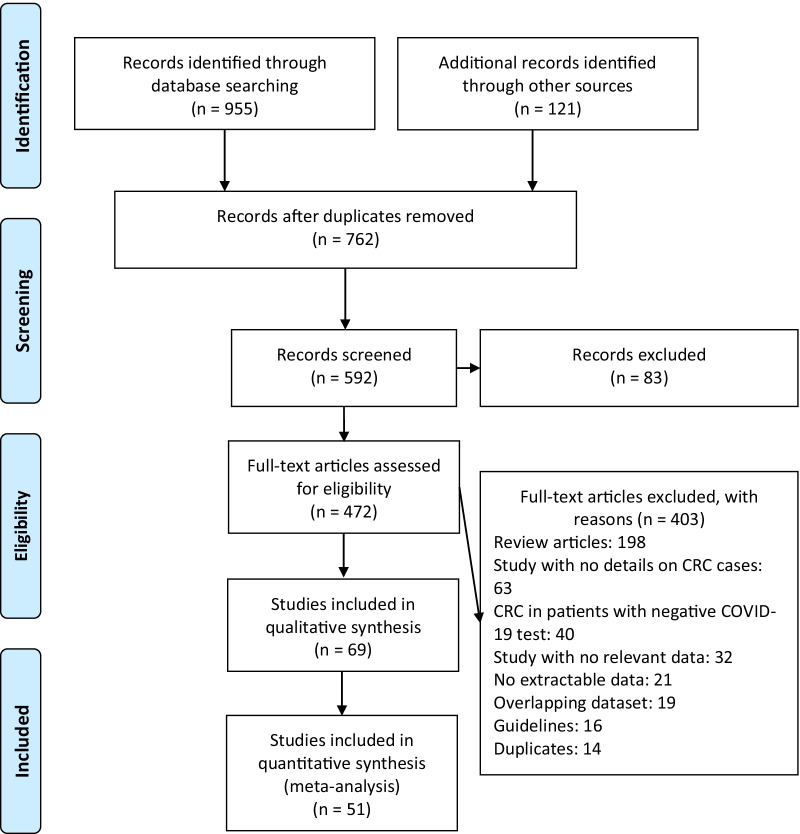
Flow diagram of literature search and data extraction from studies included in the systematic review and meta-analysis

A total of 1076 publications were identified (Fig. 2). After scanning titles and abstracts, we discarded 314 duplicate articles. Another 83 irrelevant articles were excluded based on the titles and abstracts. The full texts of the 472 remaining articles were reviewed, and 403 irrelevant articles were excluded. As a result, we identified 69 studies that met our inclusion criteria and reported SARS-CoV-2 infection in CRC patients [8–17, 23, 25–36, 43–85]. The detailed characteristics of the included studies are shown in Table 1. There were 16 case report [16, 32, 46, 48–50, 52, 54, 55, 59, 66, 67, 71, 75, 82, 84], 9 case series, 41 cohort [8–17, 23, 25, 26, 29, 31, 34, 43–45, 51, 53, 56–58, 60–65, 68–70, 73, 74, 77, 79, 80, 83, 85], 2 cross-sectional [72, 76] and 1 case–control [81] studies. These studies were conducted in China (*n* = 15), Italy (*n* = 8), United States (*n* = 6), United Kingdom (*n* = 6), Spain (*n* = 5), India (*n* = 4), France (*n* = 3), Turkey (*n* = 2), Brazil (*n* = 2), Japan (*n* = 2), Colombia (*n* = 1), Philippines (*n* = 1), Poland (*n* = 1), Iran (*n* = 1), The Netherlands (*n* = 1), Belgium (*n* = 1), United Arab Emirates (*n* = 1), Mexico (*n* = 1), Sweden (*n* = 1), Austria (*n* = 1), Hungary (*n* = 1), Australia (*n* = 1), and Chile (*n* = 1). Few studies were made within multi-countries (*n* = 3) [9, 12, 51]. The majority of the studies were single centre [11, 16, 23, 25, 27, 28, 30–35, 44–50, 52, 54, 55, 58, 59, 61–63, 65–69, 71, 72, 74–76, 78, 80, 82–84] and only 25 studies were multi-centre [8–10, 12–17, 26, 29, 36, 43, 51, 53, 56, 57, 60, 64, 70, 73, 77, 79, 81, 85]. The median NOS score for these studies was 6 (range, 5–7). Among the 69 included studies, 64 studies were moderate-quality studies (i.e., NOS scores were between 5 and 7) and 5 studies demonstrated a relatively high quality (i.e., NOS scores > 7); Table 1.

### Meta-analysis of SARS-CoV-2 infection in patients with CRC

**Fig. 3:**
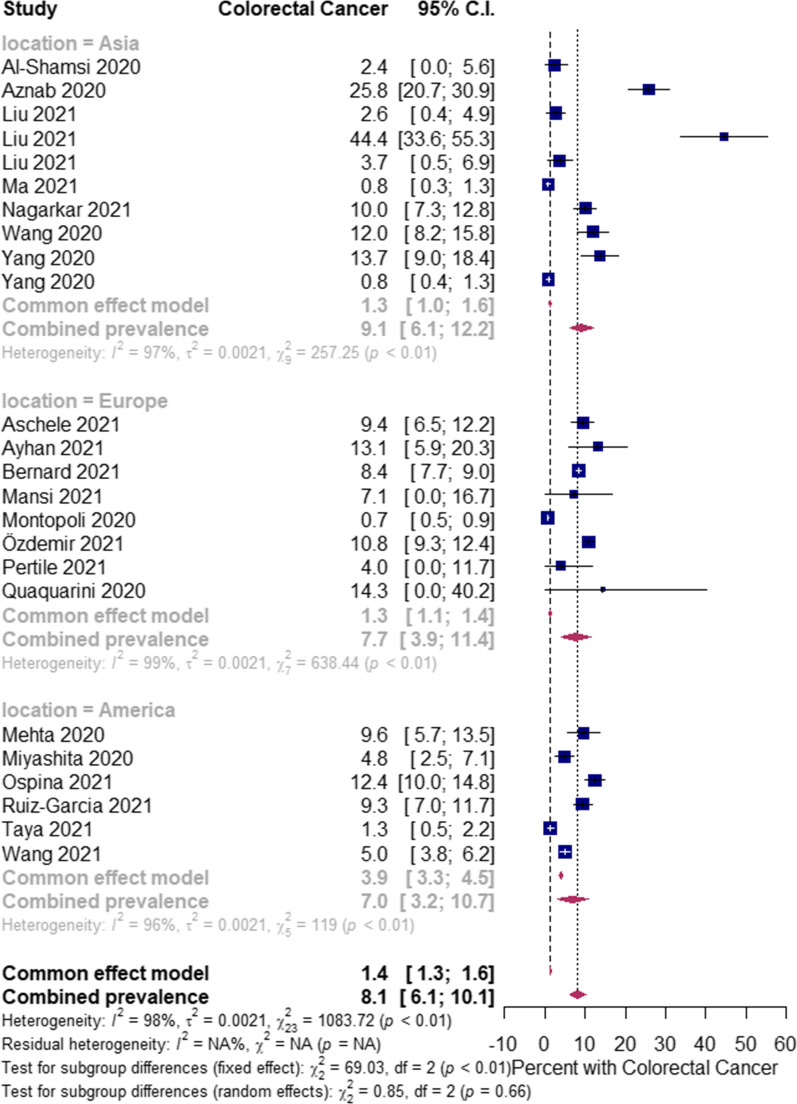
Pooled estimate for the prevalence of community-acquired COVID-19 infection in colorectal cancer patients stratified by the study location type

**Fig. 4:**
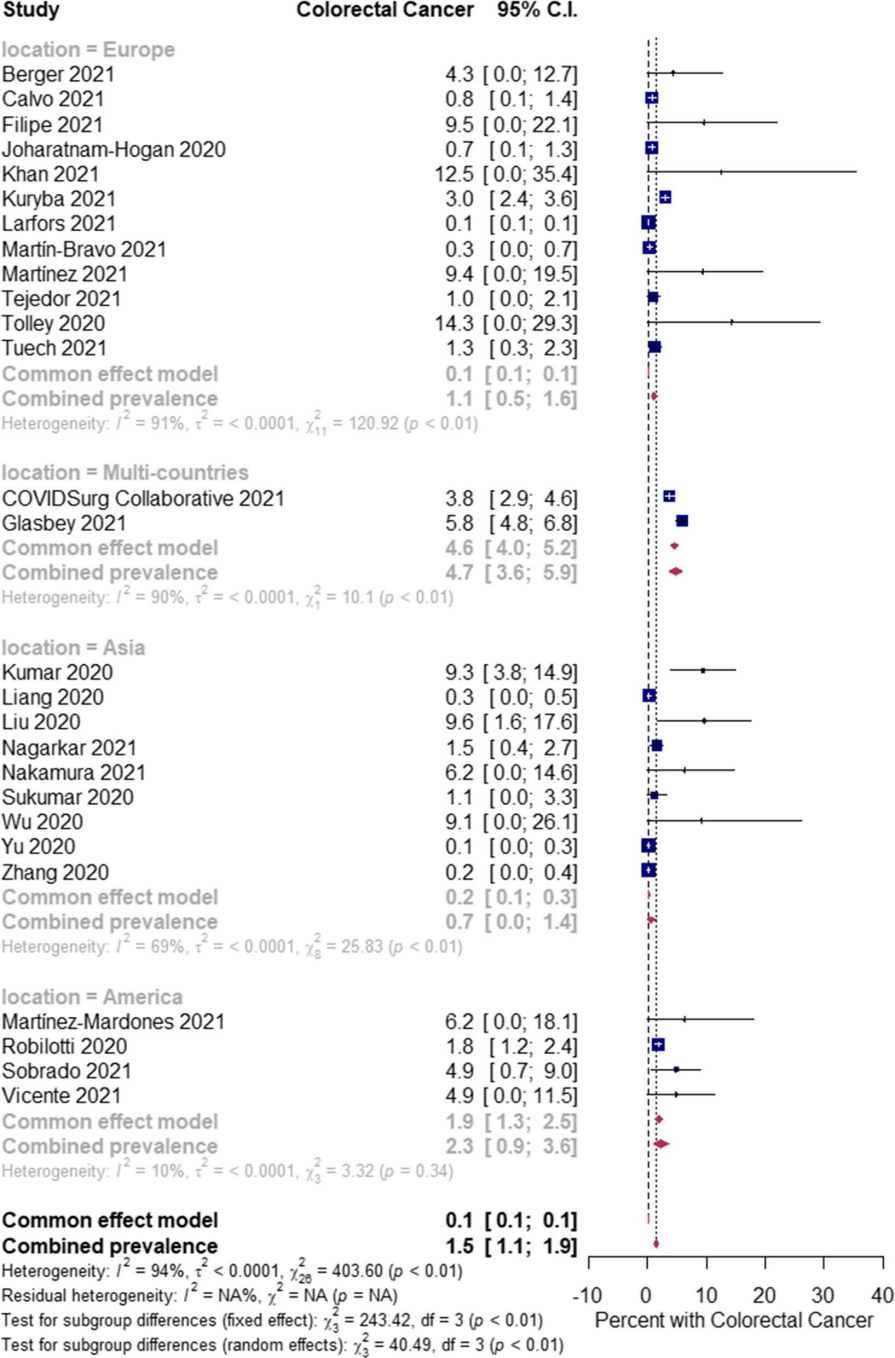
Pooled estimate for the prevalence of hospital-acquired COVID-19 infection in colorectal cancer patients stratified by the study location type

The overall pooled proportions of CRC patients who had laboratory-confirmed community-acquired and hospital-acquired SARS-CoV-2 infections were 8.1% (95% CI 6.1 to 10.1, *n* = 1308, 24 studies, *I*^*2*^ 98%, *p* = 0.66), and 1.5% (95% CI 1.1 to 1.9, n = 472, 27 studies, *I*^*2*^ 94%, *p* < 0.01), respectively; (Fig. 3, Fig. 4).

In community-acquired infected SARS-CoV-2 patients, subgroup analysis showed some difference in the rates between all patients (Asia, Europe and America groups) [8, 11, 13–17, 23, 28, 29, 33, 34, 43–45, 58, 62–65, 70, 76, 81, 86]; and the Asia group [(9.1% (95% CI 6.1 to 12.2, *n* = 252, 10 studies, *I*^*2*^ = 97%)] [11, 15–17, 23, 28, 45, 58, 65, 86]; Europe group [(7.7% (95% CI 3.9 to 11.4, *n* = 801, 8 studies, *I*^*2*^ = 99%)] [8, 14, 29, 33, 34, 43, 44, 64]; and America group [7.0% (95% CI 3.2 to 10.7, *n* = 229, 6 studies, *I*^*2*^ = 96%)] [13, 62, 63, 70, 76, 81], respectively; Fig. 3. In the hospital-acquired SARS-CoV-2 infected patients, subgroup analysis showed a significant difference in the rates between all patients (Europe, multi-countries, Asia and America) [9, 10, 25–28, 30, 31, 35, 36, 47, 51, 53, 56, 57, 60, 61, 65, 68, 69, 72, 74, 77, 79, 80, 83, 85]; and Europe only patients [1.1% (95% CI 0.5 to 1.6, *n* = 178, 12 studies, *I*^*2*^ = 91%)] [10, 25–27, 35, 47, 53, 56, 60, 61, 77, 79]; multi-countries only patients [4.7% (95% CI 3.6 to 5.9, *n* = 212, 2 studies, *I*^*2*^ = 90%)] [9, 51]; Asia only patients [0.7% (95% CI 0.0 to 1.4, *n* = 32, 9 studies, *I*^*2*^ = 69%)] [28, 31, 36, 57, 65, 68, 74, 83, 85]; and America only patients [2.3% (95% CI 0.9 to 3.6, *n* = 32, 4 studies, *I*^*2*^ = 10%)] [30, 69, 72, 80], respectively; Fig. 4.

### Demographic and clinical characteristics of CRC patients with SARS‑CoV‑2 infection

The included studies had a total of 3362 CRC patients with confirmed SARS-CoV-2 infection as detailed in Table 1. Amongst these 3362 patients, all patients were adults. The median patient age ranged from 51.6 years to 80 years across studies. There was an increased male predominance in CRC patients diagnosed with SARS-CoV-2 in most of the studies [*n* = 2243, 66.7%] [9, 11, 14, 16, 17, 25, 27, 28, 30, 32–35, 47, 52–59, 61, 62, 71, 72, 74, 75, 77, 81–85] and majority of the patients belonged to White (Caucasian) (*n* = 262, 7.8%), Hispanic (*n* = 156, 4.6%) and Asian (*n* = 153, 4.5%) ethnicity [11, 13–17, 25–34, 36, 43, 44, 46–50, 52–55, 57–61, 67, 70–73, 75–78, 80, 81, 83–85]. Most patients were diagnosed for CRC through symptoms, endoscopy, radiological imaging, biopsies and tumor markers [8–10, 12–14, 16, 17, 23, 25–36, 43, 44, 46–56, 58–61, 65–77, 80–85]. The main source of SARS-CoV-2 infection in CRC patients was community-acquired (*n* = 2882, 85.7%; *p* = 0.014) [8, 11–17, 23, 28, 29, 33, 34, 43–45, 48, 50, 52, 55, 58, 59, 62–67, 70, 71, 75, 76, 81–83]. Most of those SARS-CoV-2 patients had stage III CRC (*n* = 725, 21.6%; *p* = 0.036) [9, 13, 17, 35, 45, 72, 83]; and were treated mainly with surgical resections (*n* = 304, 9%) and chemotherapies (*n* = 187, 5.6%), *p* = 0.008 [9–11, 16, 17, 23, 25, 26, 28, 29, 31–36, 43–45, 47, 49, 51, 53, 54, 56–58, 60, 61, 68, 72, 74, 77, 80, 82, 85]. The most common tumor symptoms patients experienced were change in bowel habits (*n* = 26, 0.8%), diarrhoea (*n* = 25, 0.7%), abdominal pain (*n* = 23, 0.7%), and nausea and vomiting (*n* = 21, 0.6%); *p* = 0.048 [11, 16, 17, 27, 46, 50, 55, 58, 61, 70, 72, 81]. Many of the CRC patients infected with COVID-19 had pre-existing hypertension (*n* = 68, 2%) and/or diabetes mellitus (*n* = 49, 1.4%), *p* = 0.027 [11, 17, 28, 31, 33, 34, 44, 46, 55, 57, 58, 61, 62, 71, 72, 74, 75, 77, 79, 80, 83, 85].

### Patient treatment outcome and predictors of mortality

Patients were stratified based on treatment outcome (mortality or survival). A summary of the demographic, source of SARS-CoV-2 infection, CRC staging, treatment received, symptoms of tumor, comorbidities and medical complications with regards to final treatment outcome in 2787 patients who had either survived (*n* = 2056) or died (*n* = 731) is shown in Table 2.

**Table 2:** Demographic data of the SARS-CoV-2 patients with colorectal cancer, stratified by treatment outcome (*n* = 69 studies), 2020–2021

Variable	Findings^b^			
	All (*n* = 3362)	Survived (*n* = 2056)	Died (*n* = 731)	*p-*value^c^
*Age (years)*				
< 60	126 (3.7)	1990 (86.8)	5 (0.7)	0.000*
≥ 60	1126 (33.5)	106 (5.1)	664 (90.8)	
*Gender*				
Female	236 (7.0)	151 (7.3)	17 (2.3)	0.000*
Male	2243 (66.7)	58 (2.8)	50 (6.8)	
*Ethnicity*				
White (Caucasian)	262 (7.8)	185 (9)	20 (2.7)	0.011*
Hispanic	156 (4.6)	69 (3.3)	31 (4.2)	
Asian	153 (4.5)	117 (5.7)	37 (5.1)	
Persian	72 (2.1)	71 (3.4)	1 (0.1)	
Indian	65 (1.9)	12 (0.6)	0	
Black^a^	4 (0.12)	1 (0.05)	0	
Arab	2 (0.06)	2 (0.1)	0	
*Source of SARS-CoV-2 infection*				
Community-acquired	2882 (85.7)	1932 (94)	647 (88.5)	0.014*
Hospital-acquired	480 (14.3)	124 (6)	84 (11.5)	
*Colorectal cancer staging*				
Stage I	524 (15.6)	134 (6.5)	4 (0.5)	0.036*
Stage II	507 (15.1)	51 (2.5)	2 (0.2)	
Stage III	725 (21.6)	66 (3.2)	17 (2.3)	
Stage IV	227 (6.7)	39 (1.9)	61 (8.3)	
*Treatment*				
Surgical resections	304 (9.0)	53 (2.6)	21 (2.9)	0.008*
Chemotherapies	187 (5.6)	111 (5.4)	39 (5.3)	
Antibiotics	53 (1.6)	46 (2.2)	7 (0.9)	
Antivirals	49 (1.4)	54 (2.6)	5 (0.7)	
Colectomies	46 (1.4)	12 (0.6)	2 (0.3)	
Monoclonal antibodies	43 (1.3)	26 (1.3)	1 (0.1)	
Steroids	29 (0.9)	21 (1)	4 (0.5)	
Surgeries (nonspecific)	24 (0.7)	19 (0.9)	3 (0.4)	
Conservative (no treatment)	22 (0.6)	19 (0.9)	3 (0.4)	
Targeted therapies	20 (0.6)	3 (0.1)	2 (0.3)	
Immunotherapies	17 (0.5)	8 (0.4)	3 (0.4)	
Radiotherapy	12 (0.3)	10 (0.5)	2 (0.3)	
Stoma creation	10 (0.3)	2 (0.1)	1 (0.1)	
Immunoglobulin G	10 (0.3)	8 (0.4)	2 (0.3)	
Hartmann’s procedure	9 (0.3)	2 (0.1)	1 (0.1)	
Sigmoidectomies	8 (0.2)	7 (0.3)	1 (0.1)	
Hydroxychloroquine	6 (0.2)	5 (0.2)	1 (0.1)	
Palliative treatment	5 (0.2)	4 (0.2)	1 (0.1)	
Red blood cell transfusion	3 (0.1)	2 (0.1)	1 (0.1)	
Ileostomy	3 (0.1)	3 (0.1)	0	
Colostomy	3 (0.1)	3 (0.1)	0	
Hormones	3 (0.1)	–	–	
Stoma closure	2 (0.06)	2 (0.1)	0	
Stents	2 (0.06)	–	–	
Anastomosis	2 (0.06)	2 (0.1)	0	
Antifungals	1 (0.02)	1 (0.05)	0	
Tranexamic acid	1 (0.02)	1 (0.05)	0	
Interferon beta-1b	1 (0.02)	1 (0.05)	0	
Colonic decompression	1 (0.02)	1 (0.05)	0	
*Symptoms from the tumor*				
Change in bowel habits	26 (0.8)	10 (0.5)	7 (0.9)	0.048*
Diarrhoea	25 (0.7)	18 (0.9)	5 (0.7)	
Abdominal pain	23 (0.7)	5 (0.2)	2 (0.3)	
Nausea and vomiting	21 (0.6)	6 (0.3)	4 (0.5)	
Anaemia (unexplained iron deficiency)	8 (0.2)	7 (0.3)	1 (0.1)	
Haematochezia (blood per anus)	6 (0.2)	5 (0.2)	1 (0.1)	
Weight loss	5 (0.05)	4 (0.2)	1 (0.1)	
Blood per rectum	4 (0.1)	2 (0.1)	2 (0.2)	
Melena (black tarry stools)	3 (0.1)	2 (0.1)	1 (0.1)	
Constipation	1 (0.03)	1 (0.05)	0	
*Comorbidities*				
Hypertension	68 (2)	51 (2.5)	14 (1.9)	0.027*
Diabetes mellitus	49 (1.4)	25 (1.2)	19 (2.6)	
COPD	11 (0.3)	5 (0.2)	3 (0.4)	
Coronary heart disease	11 (0.3)	6 (0.3)	2 (0.3)	
Cerebrovascular disease	5 (0.1)	2 (0.1)	3 (0.4)	
Chronic kidney disease	4 (0.1)	2 (0.1)	2 (0.3)	
Chronic renal failure	3 (0.09)	–	2 (0.3)	
Cardiovascular disease	3 (0.09)	2 (0.1)	1 (0.1)	
Morbid obesity	3 (0.09)	2 (0.1)	1 (0.1)	
Heart failure	2 (0.06)	1 (0.05)	1 (0.1)	
Acute ischemic stroke	2 (0.06)	0	2 (0.3)	
Dyslipidemia	2 (0.06)	1 (0.05)	1 (0.1)	
Hepatitis B virus	2 (0.06)	1 (0.05)	1 (0.1)	
Hepatitis C virus	2 (0.06)	1 (0.05)	1 (0.1)	
Lynch Syndrome	2 (0.06)	2 (0.1)	0	
Congestive heart failure	2 (0.06)	0	2 (0.3)	
Asthma	1 (0.03)	1 (0.05)	0	
Cardiac arrest	1 (0.03)	1 (0.05)	0	
Chronic anaemia	1 (0.03)	1 (0.05)	0	
Diabetic ketoacidosis	1 (0.03)	1 (0.05)	0	
Tuberculosis	1 (0.03)	0	1 (0.1)	
*Complications and treatment outcomes*				
Patient was admitted to ICU	153 (4.5)	7 (0.3)	96 (13.1)	0.000*
Patient was intubated and on mechanical ventilation during the ICU stay	51 (1.5)	3 (0.1)	47 (6.4)	0.000*
Patient experienced acute respiratory distress syndrome	36 (1.1)	1 (0.05)	29 (4)	0.000*

*COPD* Chronic obstructive pulmonary disease, *ICU* Intensive care unit, *SARS-CoV-2* Severe acute respiratory syndrome coronavirus 2

^a^Patients with black ethnicity include African-American, Black African, African and Afro-Caribbean patients

^b^Data are presented as number (%)

^c^Chi-square (*χ*^*2*^) test was used to compare between survival and death groups

Percentages do not total 100% owing to missing data

* Represents significant differences

Those patients who died were more likely to have been older in age (≥ 60 years old: 90.8% vs 0.7%; *p* = 0.000); and more likely to be men [male gender: 6.8% vs 2.3%; *p* = 0.000]. Majority of patients who died had an Asian (n = 37, 5.1%) and Hispanic ethnicity (*n* = 31, 4.2%; *p* = 0.011). CRC patients who transmitted SARS-CoV-2 from the community had a higher mortality compared to those patients who acquired the SARS-CoV-2 infection from a hospital source (88.5% vs 11.5%; *p* = 0.014). As expected with the CRC stating, patients with advanced stage had a high mortality [death in stage IV CRC patients occurred in *n* = 61 (8.3%), *p* = 0.036]. CRC patients infected with SARS-CoV-2 who received chemotherapy had about two-fold increased risk of mortality compared to CRC patients with SARS-CoV-2 who had surgical resections (39 (5.3%) vs 21 (2.9%); *p* = 0.008). The most common tumor symptoms in CRC patients with SARS-CoV-2 infection in whom mortality was reported were the change in bowel habits (*n* = 7, 0.9%) and diarrhoea (*n* = 5, 0.7%); *p* = 0.048. Patients with a pre-existing diabetes mellitus (*n* = 19, 2.6%) and hypertension (*n* = 14, 1.9%) had the highest mortality rate compared to other comorbidities; *p* = 0.027. Mortality rate was significantly very high in CRC patients infected with SARS-CoV-2 who were admitted to the intensive care unit (0.3% vs 13.1%; *p* = 0.000), placed on mechanical ventilation (0.1% vs 6.4%; *p* = 0.000) and/or suffered acute respiratory distress syndrome (0.05% vs 4%; *p* = 0.000).

**Fig. 5:**
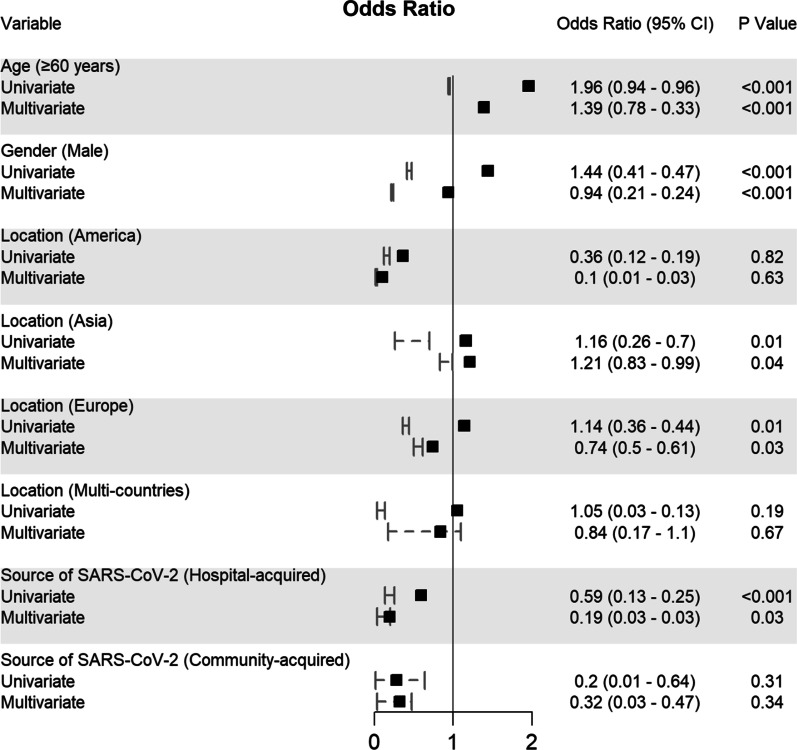
Predictors of mortality in patients hospitalized for colorectal cancer and SARS-CoV-2 (*n* = 2768)

**Fig. 6:**
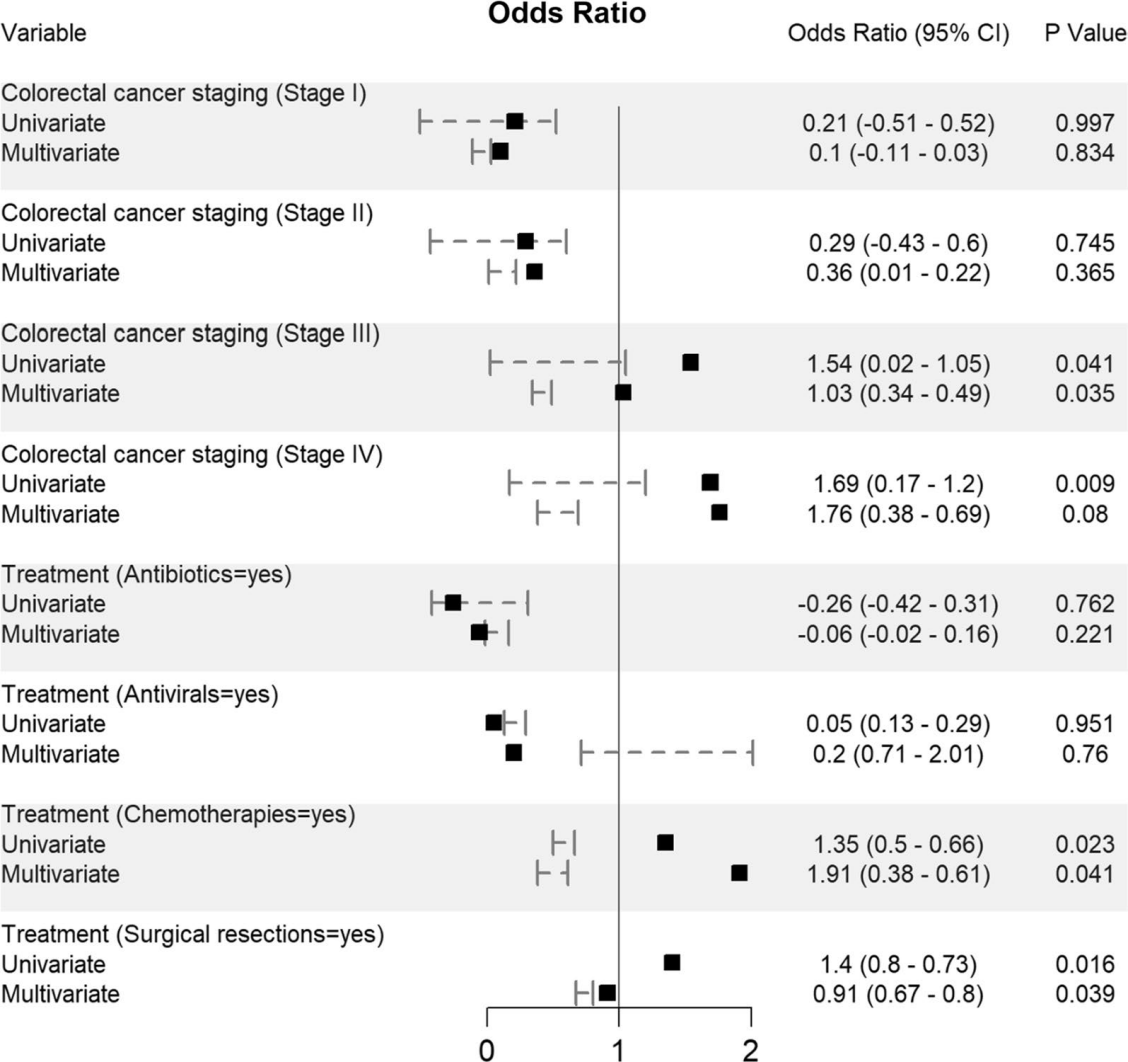
Predictors of mortality in patients hospitalized for colorectal cancer and SARS-CoV-2 (*n* = 2768)

**Fig. 7:**
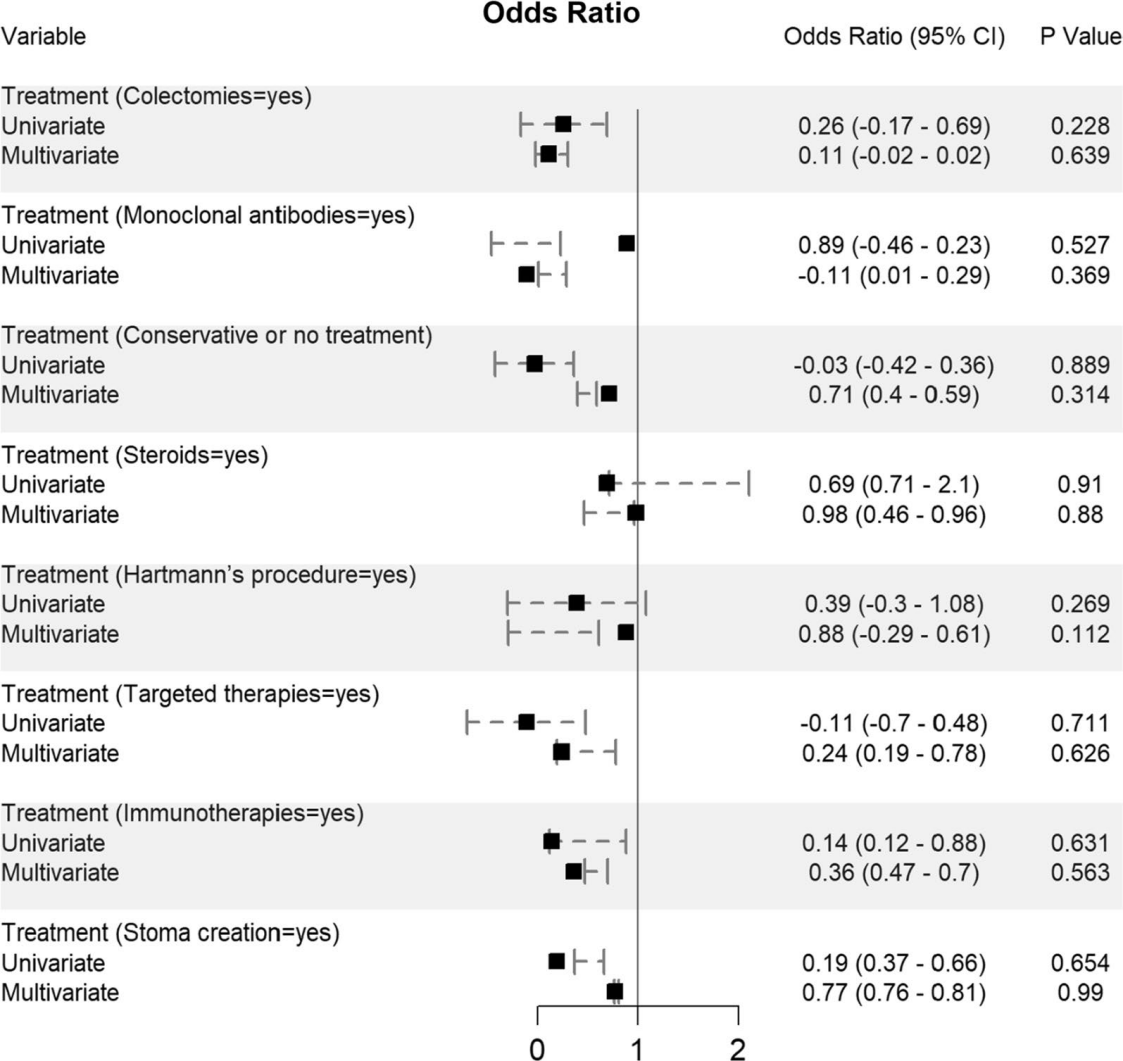
Predictors of mortality in patients hospitalized for colorectal cancer and SARS-CoV-2 (n = 2768)

**Fig. 8:**
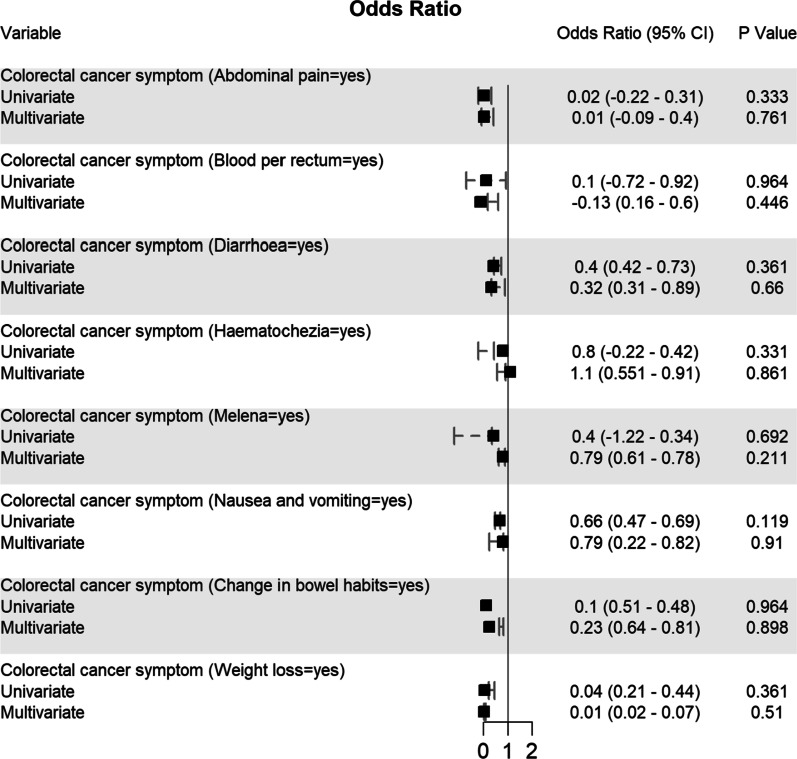
Predictors of mortality in patients hospitalized for colorectal cancer and SARS-CoV-2 (*n* = 2768)

**Fig. 9:**
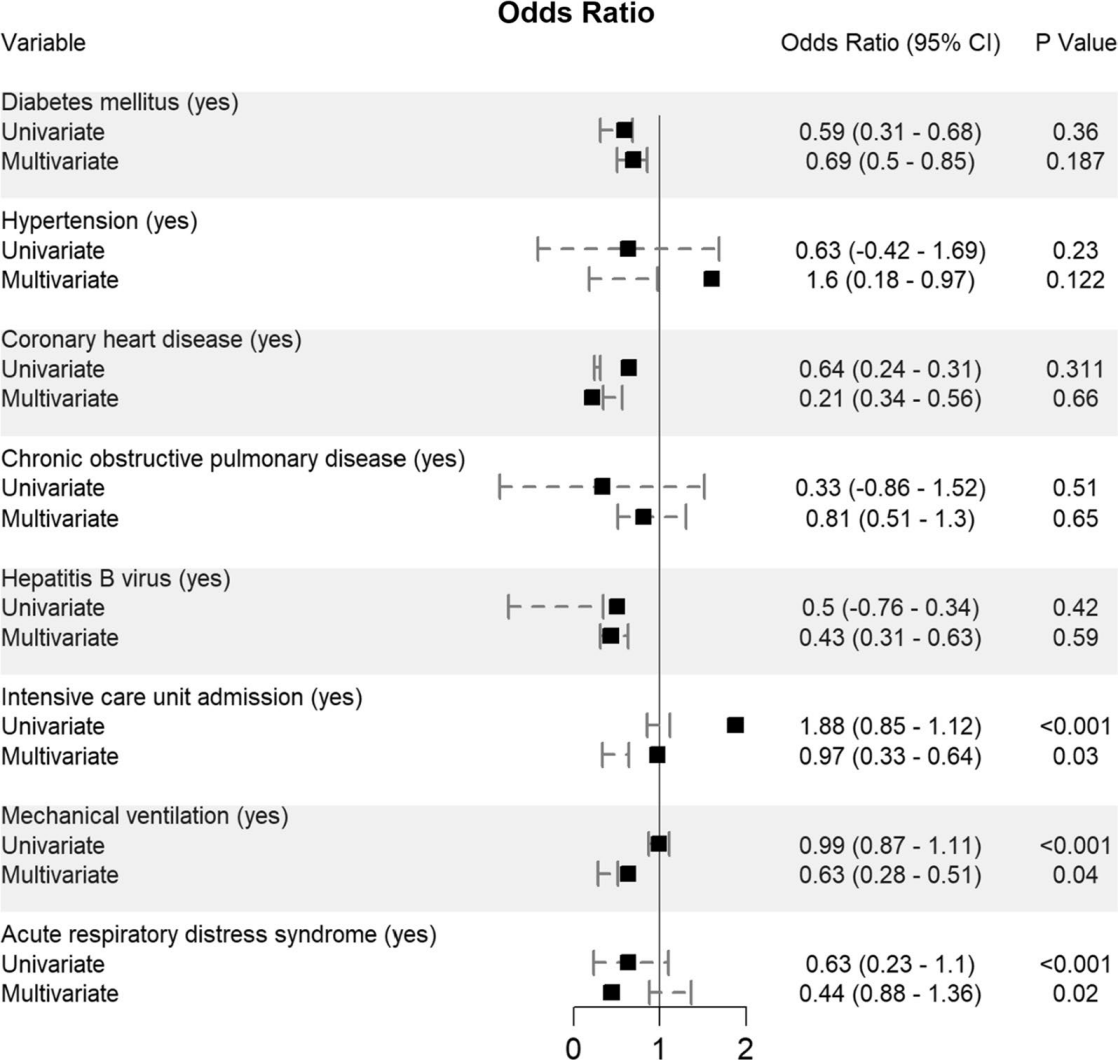
Predictors of mortality in patients hospitalized for colorectal cancer and SARS-CoV-2 (*n* = 2768)

Potential determining variables associated in survival and death groups were analysed through binary logistic regression analysis and shown in Fig. 5, Fig. 6, Fig. 7, Fig. 8 and Fig. 9. As expected, old age (≥ 60 years) (OR 1.96, 95% CI 0.94–0.96; *p* < 0.001), male gender (OR 1.44, 95% CI 0.41–0.47; *p* < 0.001), CRC patients infected with SARS-CoV-2 who came from Asia (OR 1.16, 95% CI 0.26–0.7; *p* = 0.01) and Europe (OR 1.14, 95% CI 0.36–0.44; *p* = 0.01), or transmitted the SARS-CoV-2 viral infection from a hospital source (OR 0.59, 95% CI 0.13–0.25; *p* < 0.001) are associated with increased odd ratio for death; Fig. 5. Among the CRC staging groups, patients who were infected with SARS-CoV-2 and presented with CRC stage III (OR 1.54, 95% CI 0.02–1.05; *p* = 0.041) and stage IV (OR 1.69, 95% CI 0.17–1.2; *p* = 0.009) had a high OR of death; Fig. 6. The odd ratios of death were also high in CRC patients infected with SARS-CoV-2 who had chemotherapy (OR 1.35, 95% CI 0.5–0.66; *p* = 0.023) and surgical resections (OR 1.4, 95% CI 0.8–0.73; *p* = 0.016); Fig. 6. Other predictors for increased risk of succumbing included admission to intensive care unit (OR 1.88, 95% CI 0.85–1.12; *p* < 0.001), intubation and placing on mechanical ventilation (OR 0.99, 95% CI 0.87–1.11; *p* < 0.001), and suffering from acute respiratory distress syndrome (OR 0.63, 95% CI 0.23–1.1; *p* < 0.001); Fig. 9.

These variables were considered needing further evaluation and, thus, were included in multivariate regression analysis. Nevertheless, multivariate analysis confirmed old age (≥ 60 years), male gender, CRC patients with SARS-CoV-2 infection located in Asia and Europe, who transmitted SARS-CoV-2 from hospital, CRC stage III, who had chemotherapy and surgical resections, admitted to intensive care unit, intubated and placed on mechanical ventilation and suffered acute respiratory distress syndrome were significantly associated with increased death. Although univariate analysis showed CRC stage IV patients with SARS-CoV-2 infection was significantly associated with increased mortality (*p* = 0.009), however, this finding was not reciprocated by multivariate analysis; Fig. 5.

## Discussion

In this large systematic review and meta-analysis, we included 3362 patients with laboratory-confirmed SARS-CoV-2 infection from 69 observational studies in order to estimate the prevalence of COVID-19 disease in CRC patients. A better understanding of the prevalence of SARS-CoV-2 disease in CRC patients allows the development of more specific and more efficient ways of prevention and therapy. As expected, overall prevalence of community-acquired SARS-CoV-2 infection in CRC patients was fivefold higher compared to hospital-acquired SARS-CoV-2 infection in this group of cancer patients (8.1% vs 1.5%). This could be chiefly explained by the maintenance of good knowledge and compliance of infection prevention and control by healthcare providers [87], antimicrobial stewardship [88], and robust surveillance for hospital-acquired infections and antimicrobial resistance [89] within healthcare organizations that provide healthcare for CRC patients. Prevalence of SARS-CoV-2 infection acquired from the community in CRC patients was almost similar in Asia (9.1%, 95% CI 6.1–12.2), Europe (7.7%, 95% CI 3.9–11.4), and America (7.0%, 95% CI 3.2–10.7). However, SARS-CoV-2 infection rate acquired from the hospital in CRC patients was the highest in studies conducted in multiple countries (4.7%, 95% CI 3.6–5.9). In general, there is an approximately ninefold variation in CRC prevalence rates by world regions, with the highest rates in European regions, Australia/New Zealand, and Northern America; and rates of CRC prevalence tend to be low in most regions of Africa and in South Central Asia [90]. However, negative impact of SARS-CoV-2 infection on CRC patients should be considered as the COVID-19 pandemic has led to a sustained reduction in the number of people referred, diagnosed, and treated for CRC [22, 91–93]. The findings in this meta-analysis showed different results from previous systematic meta-analyses that evaluated SARS-CoV-2 infection among CRC patients [24, 94]. We reported a much lower prevalence of SARS-CoV-2 infection in CRC patients [3.43%] compared to the previous two systematic meta-analyses [45.1% and 20.5%, respectively] [24, 94]. The current meta-analysis is more comprehensive and included a total of 69 studies [8–17, 23, 25–36, 43–85] including a total of 3362 patients; whose details on final treatment outcome were available; in comparison to smaller sample size in previous meta-analyses (sample size: *n* = 92 and *n* = 20, respectively) [24, 94]. The inclusion of 65 recently published studies [8–10, 12–17, 23, 25–36, 38, 43, 44, 46–56, 58–84] contributed to the refinement on evidence of the demographic and clinical characteristics; in addition to final treatment outcome in CRC patients with SARS-CoV-2 illness.

We report no paediatric case with SARS-CoV-2 infection and CRC as the incidence of CRC is rare compared with that in adults (prevalence of CRC in patients under age 20 was reported to be 0.2%) [95]. Unlike in adults, familial cancer history is not strongly associated with CRC in children [96]. The lack of childhood cases with COVID-19 and CRC in our review can also be justified by the fact that most children with SARS-CoV-2 disease have mild symptoms or have no symptoms at all [97] and the high severity of COVID-19 tends to be much lower in children compared to adults [98]. However, CRC is more likely to be lethal in children and young adults than middle-aged adults and was explained by the higher incidence of precancerous diseases (such as polyposis, colitis) and mucinous adenocarcinoma and/or late CRC diagnosis in children [95, 96]. Hence CRC is usually diagnosed later and potentially associated with worst prognosis in young groups [95, 96], detecting CRC at an early, more treatable stage is important for cure and survival.

In our review, males gender predominated development of SARS-CoV-2 illness in CRC patients, a finding suggested in most of the reports [9, 11, 14, 16, 17, 25, 27, 28, 30, 32–35, 47, 52–59, 61, 62, 71, 72, 74, 75, 77, 81–85] and in contradiction with data from other reports suggesting an equal proportion of COVID-19 cases in CRC patients for both genders [14, 23, 29, 31, 60, 80] or patients with CRC and SARS-CoV-2 illness were mostly females [11, 36, 46, 49, 50, 66, 67, 76]. This review reflects previous studies in showing that the overall incidence of CRC is higher in males than in females [99–101]. This increased vulnerability of men to developing CRC may be due to a number of biological and gender-related (behavioural) factors [99, 102–104]. Men are more likely to have a diet high in red and processed meat [105], be heavier consumers of alcohol [106], and more likely to smoke [107]. Men also have a greater propensity to deposit visceral fat [108, 109] which is associated with increased risk of CRC [99–101, 110]. Moreover, SARS-CoV-2 has been known to infect cells via angiotensin-converting enzyme 2 receptors for entry which have been found to be highly expressed in human males and the angiotensin-converting enzyme 2 receptor gene is X-linked [111, 112]. However, male excess in CRC in our review might be attributed mainly to the differences in the inclusion criteria and the population age groups included in the studies; or can be explained by higher rates of comorbidities among men [113, 114], higher trend among females to follow hand hygiene and preventive care [115, 116], stronger immune response to infections in females who outlive men [117] or lower rates of healthcare service utilization by males [118].

We found development of COVID-19 in CRC patients was highest in people of White (Caucasian) [14, 35, 43, 44, 47, 53, 76, 77], Hispanic [13, 70, 72, 80] and Asian ethnicity [11, 15–17, 28, 57, 58, 83] (7.8%, 4.6% and 4.5%, respectively). Moreover, we found mortality rate in CRC patients infected with COVID-19 was significantly high in patients with Asian and Hispanic ethnicity [5.1% and 4.2%, *p* = 0.011]. CRC is a substantial public health burden and it is increasingly affecting populations in Asian and Hispanic countries [119, 120]. The risk of contracting COVID-19 in people with Asian and Hispanic ethnicity is known to be high and clinical prognosis in those people has been previously described to be poor [121, 122]. CRC screening has been playing an important role in reducing its disease burden [123]. The surveillance system in countries with high burden needed to provide facilities for CRC screening and public awareness education program should be considered in national and international planes to increases the self-participation of people [124]. Financial limitation and lack of authorities are still the main obstacles in the way of CRC screening in most Asian and Hispanic countries with low-income status [125, 126]. Because most of the studies included in our review that reported the ethnicity of CRC cases infected with COVID-19 were either from China, Italy, United States of America, or United Kingdom; representation of other ethnicities at risk to develop COVID-19 in CRC patients can be misleading. For instance, we report a very low prevalence of SARS-CoV-2 infection in CRC patients in Black population (*n* = 4, 0.12%), yet, in the United States, the incidence and mortality rates for CRC are higher among Black patients, particularly men, than among those in other racial or ethnic groups, and, among Black patients, CRC occurs at a higher rate below age 50 years [127].

During the COVID-19 pandemic, increasing age in combination with male gender might denote seriously sick patients who can potentially have more morbidity and propensity to die [128, 129]. The majority of CRC patients hospitalized with SARS-CoV-2 are older and seemed to have underlying medical conditions [11, 27, 29, 31, 33, 35, 47, 57, 59, 60, 62, 72, 77, 85], with increased age being associated with clinical severity, including case fatality. Furthermore, comorbidities [11, 16, 17, 27, 28, 31, 33, 46, 57, 58, 62, 72, 77, 83, 85] and advanced CRC stages (stage III and IV) [26, 27, 29, 31, 33, 35, 36, 46–49, 60, 72, 77] affect the prognosis of COVID-19. Although chemotherapies and surgical resections are the primary treatment modalities for early stage CRC (stage I through III) [130, 131], we report active treatment of both chemotherapies and surgical resections were associated with an increased risk for severe disease and death from COVID-19 in CRC patients, a finding which is in line with previous meta-analyses [132, 133]. Although one meta-analysis found chemotherapy was associated with an increased risk of death from COVID-19 in patients with cancer but failed to show any significant association between other treatments like surgery due to the very small number of included studies [132], our meta-analysis shown the possible increase in risks of severe COVID-19 and death in SARS-CoV-2-infected CRC patients receiving surgical resections which is in consistent with recent cohort and meta-analysis studies [133–135]. Chemotherapies commonly used to treat cancer, including CRC, affect not only the tumor but also the immune system [136]. Advanced COVID-19 syndrome is characterized by the uncontrolled and elevated release of pro-inflammatory cytokines and suppressed immunity, leading to the cytokine storm [137]. An impaired immune system might cause a decreased inflammatory response against SARS-CoV-2 and, thus, protecting from cytokine storm [138]. The uncontrolled and dysregulated secretion of inflammatory and pro-inflammatory cytokines in SARS-CoV-2 patients with CRC is positively associated with the severity of the viral infection and mortality rate and this cascade of events may lead to multiple organ failure, ARDS, or pneumonia and need for ICU admission and mechanical ventilation [137, 139]. Furthermore, postoperative pulmonary complications was reported to occur in half of patients with perioperative SARS-CoV-2 infection and are associated with high mortality [135], therefore, consideration should be given for postponing non-critical procedures and promoting nonoperative treatment in CRC patients to delay or avoid the need for surgery [140]. When hospitals recommence routine surgical treatments, this will be in hospital environments that remain exposed to SARS-CoV-2, so strategies should be developed to reduce in-hospital SARS-CoV-2 transmission and mitigate the risk of postoperative complications in CRC patients [135].

### Limitations

First, while most of the evidence discussed were based on many cohorts, case reports, case-series and few cross-sectional and case–control studies, many of these are small and not necessarily generalizable to the current COVID-19 clinical environment in patients with CRC history. Second, to asses factors associated with mortality, larger cohort of patients is needed. Last, almost all studies included in this review were retrospective in design which could have introduced potential reporting bias due to reliance on clinical case records.

## Conclusion

Patients with CRC are at increased risk of severe complications from SARS-CoV-2 which may include ARDS, or pneumonia and need for ICU admission and mechanical ventilation. Key determinants that lead to increased mortality in CRC patients infected with COVID-19 include older age (≥ 60 years old); male gender; Asian and Hispanic ethnicity; if SARS-CoV-2 was acquired from hospital source; advanced CRC (stage III and IV); if patient received chemotherapies or surgical treatment; and if patient was admitted to ICU, ventilated or experienced ARDS.

## Data Availability

All data generated or analysed during this study are included in this published article except for the datasets generated and analysed to explore the effect of various demographic parameters and clinical variables on patient’s final treatment outcome. These datasets are not publicly available due privacy concern but will be available, please contact the corresponding author for data requests.

## Abbreviations

ARDS: Acute respiratory distress syndrome
COVID-19: Coronavirus disease 2019
CRC: Colorectal cancer
ICU: Intensive care unit
NOS: Newcastle–Ottawa scale
PRISMA: Preferred reporting items for systematic reviews and meta-analyses
SARS-CoV-2: Severe acute respiratory syndrome coronavirus 2
